# Efficacy and safety of tyrosine kinase inhibitor combination therapy for glioblastoma: a meta-analysis with trial sequential analysis of randomized controlled trials

**DOI:** 10.3389/fonc.2026.1796708

**Published:** 2026-04-20

**Authors:** Pan Wei, Jing Jiang, Ming Xiao, Yuekang Zhang

**Affiliations:** 1 Department of Neurosurgery, West China Hospital, West China Medical School, Sichuan University, Chengdu, China; 2 Department of Neurosurgery, The First People’s Hospital of Longquanyi District, Chengdu, China; 3 Department of Physical Examination, The First People’s Hospital of Longquanyi District, Chengdu, China

**Keywords:** glioblastoma, meta-analysis, overall survival, placebo, progression-free survival, tyrosine kinase inhibitor

## Abstract

**Background:**

Tyrosine kinase inhibitors (TKIs) administered as monotherapy have demonstrated limited clinical efficacy in glioblastoma (GBM). This study synthesized evidence from existing randomized controlled trials (RCTs) to evaluate the efficacy and safety of combination treatments incorporating TKIs for GBM.

**Methods:**

We performed a systematic literature search in PubMed, the Cochrane Library, Web of Science, and Embase to identify RCTs published up to January 10, 2026. The efficacy of treatments was evaluated by progression-free survival (PFS), overall survival (OS), objective response rate (ORR), stable disease (SD), and progressive disease (PD), while safety was assessed by the incidence of adverse events (AEs). Survival outcomes were pooled as hazard ratios (HRs) and dichotomous outcomes as risk ratios (RRs), each reported with 95% confidence intervals (CIs). Heterogeneity across studies was assessed using the Cochrane Q test, I² statistic, and 95% prediction intervals (PIs). Trial sequential analysis was incorporated into the meta-analysis to control the likelihood of false-positive and false-negative conclusions.

**Results:**

This analysis included 10 RCTs encompassing 1,435 GBM. The overall analysis revealed that combination therapies incorporating TKIs significantly improved PFS (HR [95% CI] = 0.834 [0.697-0.998], 95% PI: 0.506-1.376) and ORR (RR [95% CI] = 1.664 [1.083-2.558], 95% PI: 0.917-2.868) compared with TKI-free control regimens. However, no significant benefits were observed for OS, SD, or PD. Further subgroup analyses stratified by combination regimens (TKIs plus standard chemoradiotherapy or TKIs plus non-standard therapies) and by GBM status (newly diagnosed or recurrent GBM) did not identify any statistically significant efficacy differences (all *p* > 0.05). Safety analyses indicated that although TKI-containing combination therapies did not increase the overall risk of grade ≥ 3 AEs (RR [95% CI] = 1.177 [0.998-1.388], 95% PI: 0.758-1.826), they were associated with higher incidences of grade ≥ 3 neutropenia, fatigue, and diarrhea, while being associated with a reduced risk of decreased lymphocyte count (all *p* < 0.05).

**Conclusion:**

Our study suggests that combination regimens incorporating TKIs may have only modest therapeutic activity for GBM, showing signals of improved PFS and ORR but no OS benefit in the current evidence base. Clinicians should remain vigilant for AEs associated with these combination regimens, ensure close monitoring, and promptly implement appropriate measures to manage and mitigate toxicity.

**Systematic Review Registration:**

https://www.crd.york.ac.uk/prospero/, identifier CRD420261293988.

## 1 Introduction

Glioblastoma (GBM) remains the most common and lethal primary malignant tumor of the central nervous system, accounting for the majority of malignant gliomas and carrying a uniformly poor prognosis despite multimodal therapy (1–4). The current standard of care for GBM, consisting of maximal safe resection followed by radiotherapy (RT) with concomitant and adjuvant temozolomide (TMZ) (Stupp regimen) (5), provides only modest survival benefits. Median overall survival (OS) remains approximately 14–16 months, with a 5-year survival rate of around 5% (6–8). Recurrence is nearly universal, and outcomes after relapse are substantially worse, with short progression-free survival (PFS) and median survival often below one year (9–11).

Several biological challenges and practical limitations contribute to these unfavorable results. GBM is highly infiltrative, often extending beyond radiographic boundaries, which significantly limits the potential for surgical cure (12). The blood-brain barrier (BBB), along with the molecular and spatial heterogeneity of the tumour, hinders effective drug delivery and promotes therapeutic resistance (5, 13–15). Large-scale molecular profiling, notably through The Cancer Genome Atlas, has revealed recurrent alterations converging on a limited number of core pathways-primarily the receptor tyrosine kinase (RTK)/Ras/phosphoinositide 3-kinase (PI3K), p53, and retinoblastoma (Rb) signaling pathways (16). Frequent aberrations in the RTK pathway have been identified, including amplification and mutations in epidermal growth factor receptor (EGFR), platelet-derived growth factor receptor alpha (PDGFRA), and MET (17, 18). Alterations in EGFR, including the oncogenic EGFRvIII variant, are highly prevalent and biologically significant, driving tumor cell signaling, migration, and reshaping the tumor microenvironment (19–21).

These molecular discoveries have spurred significant efforts to integrate targeted therapies into clinical applications. Tyrosine kinase inhibitors (TKIs) targeting RTKs, such as EGFR and vascular endothelial growth factor receptor (VEGFR), have been transformative in other malignancies (22, 23). However, in GBM, clinical trials to date have yielded inconsistent outcomes. A prior phase III trial showed that compared with placebo plus lomustine, the pan−VEGFR TKI cediranib combined with lomustine did not produce a statistically significant improvement in PFS, OS, and objective response rate (ORR) in patients with recurrent GBM (24). Similarly, the final results of the N0877 trial demonstrated that adding the TKI dasatinib to standard chemoradiotherapy (CRT) (Stupp regimen) did not prolong PFS and OS in patients with newly diagnosed GBM (25). By contrast, a recent trial reported that the Stupp regimen plus anlotinib significantly improved PFS and ORR compared with the Stupp regimen plus placebo in newly diagnosed GBM (26).

Given the variability in trial outcomes and the pressing need to improve survival for patients with GBM, a comprehensive synthesis of randomized evidence on combination strategies involving TKIs is warranted. Therefore, we conducted a meta-analysis of randomized controlled trials (RCTs) to evaluate the efficacy and safety of TKI-based combination regimens, including TKIs combined with standard CRT (Stupp regimen) and TKIs integrated with non-standard therapeutic approaches, in patients with GBM. Our findings aim to guide future biomarker-driven research and inform the development of precision-based treatment strategies for this aggressive malignancy.

## 2 Methods

### 2.1 Study design

The present study was designed and reported in accordance with the Preferred Reporting Items for Systematic Reviews and Meta-Analyses (PRISMA) standards (27). Additionally, the study protocol received formal approval from PROSPERO, under the registration identifier CRD420261293988.

### 2.2 Search strategy

Two independent reviewers conducted systematic searches of PubMed, the Cochrane Library, Web of Science and Embase for RCTs evaluating TKI combination therapy in patients with GBM, covering each database from inception to January 10, 2026. No language or date limits were imposed, and searches were performed in accordance with Cochrane Collaboration guidance to capture both published and unpublished studies. Database-specific syntax was utilized, incorporating disease-related terms such as “glioblastoma,” “GBM,” “glioblastoma multiforme,” “grade IV glioma,” alongside treatment-related keywords including “tyrosine kinase inhibitor,” “tyrosine kinase inhibitors,” “TKIs,” “afatinib,” “cediranib,” “dasatinib,” “neratinib,” “imatinib,” “gefitinib,” “anlotinib,” “vandetanib,” “erlotinib,” “sorafenib,” “pazopanib,” “nilotinib.” To refine the search by study design, terms such as “randomized controlled trial,” “random*,” “controlled clinical trial,” “clinical trial,” and “placebo” were applied. The complete syntax used in the search strategy is provided in **Supplementary File 1**. Furthermore, reference lists of relevant publications were manually examined to identify additional eligible RCTs.

### 2.3 Inclusion and exclusion criteria

Included studies had to fulfill all of the following conditions: (1) RCTs; (2) enroll patients diagnosed with GBM at any disease stage; (3) interventions involving TKIs combined with standard CRT or non-standard treatment regimens; (4) control groups receiving either standard CRT alone (or with placebo) or non-standard therapies alone (or with placebo); and (5) reporting of at least one outcome measure, such as PFS, OS, ORR, stable disease (SD), progressive disease (PD), or any adverse events (AEs). Studies were excluded if they met any of the following: (1) single-arm or observational/non-interventional designs; (2) studies lacking required outcome data or presenting duplicate datasets; (3) control arms incorporating TKIs as part of the treatment regimen; or (4) case reports, preclinical animal studies, review articles, or correspondence/letters.

### 2.4 Data extraction and quality assessment

Two reviewers independently extracted study information. Recorded variables included first author, year of publication, trial phase, country, participants’ disease characteristics, sample size and age of the population, interventions in the experimental and control arms, and the outcomes considered in the meta-analysis. When a trial was reported in multiple publications, the most complete report was retained. PFS, OS, and ORR were specified as the primary endpoints, while SD, PD and any AEs were treated as secondary outcomes. For studies that did not report PFS or OS numerically, Kaplan-Meier curves were digitized using Engauge Digitizer v10.8 and time-to-event data were reconstructed following the methods of Tierney et al. (28, 29).

The methodological quality of the included RCTs was assessed using the modified Jadad scale (30) that examined randomization procedures, allocation concealment, blinding practices, and the reporting of participant dropouts. Trials receiving scores of 0–3 were categorized as low-quality, while those with scores of 4 or above were classified as high-quality. Any discrepancies between reviewers were settled by discussion or, if needed, by consulting a senior investigator.

### 2.5 Statistical analysis

Pooled time-to-event effects were expressed as hazard ratios (HRs) with 95% confidence intervals (CIs), while dichotomous outcomes were summarized as risk ratios (RRs) with 95% CIs. Between-study heterogeneity was quantified using the Cochrane Q test, the I^2^ statistic and 95% prediction intervals (PIs), with I^2^ > 50% taken to indicate substantial heterogeneity (31, 32). In cases where heterogeneity was not observed, a fixed-effects model was employed for result estimation. However, when heterogeneity was present, a random-effects model based on the DerSimonian and Laird approach was utilized (33). Subgroup analyses were performed based on whether TKIs were combined with standard CRT or with non−standard treatment regimens, and by GBM status (newly diagnosed versus recurrent). The stability of pooled estimates was examined by leave-one-out sensitivity analyses. Publication bias was assessed visually with funnel plots and formally with Begg’s and Egger’s tests (34, 35). All analyses were performed in R software 4.3.2 and STATA 12.0. A two-sided *p*-value of less than 0.05 was considered statistically significant.

### 2.6 Trial sequential analysis

Trial sequential analysis (TSA) was used in the meta-analysis to reduce the probability of false-positive and false-negative results (36). Survival outcomes were assessed using STATA 12.0 and R software 4.3.2 under an *a priori* information size (APIS) strategy. Dichotomous endpoints were processed in TSA v0.9.5.10 Beta, which computed the required information size (RIS) and set trial sequential monitoring boundaries. Conclusive evidence was deemed present if the cumulative Z-curve surpassed the APIS or RIS boundary, or crossed the monitoring threshold, indicating that additional studies would be unnecessary. RIS and APIS were derived using prespecified parameters: a two-sided significance level (α = 0.05), statistical power of 80% (1-β = 0.80), and an expected 15% relative risk reduction.

## 3 Results

### 3.1 Study selection

**Figure 1:**
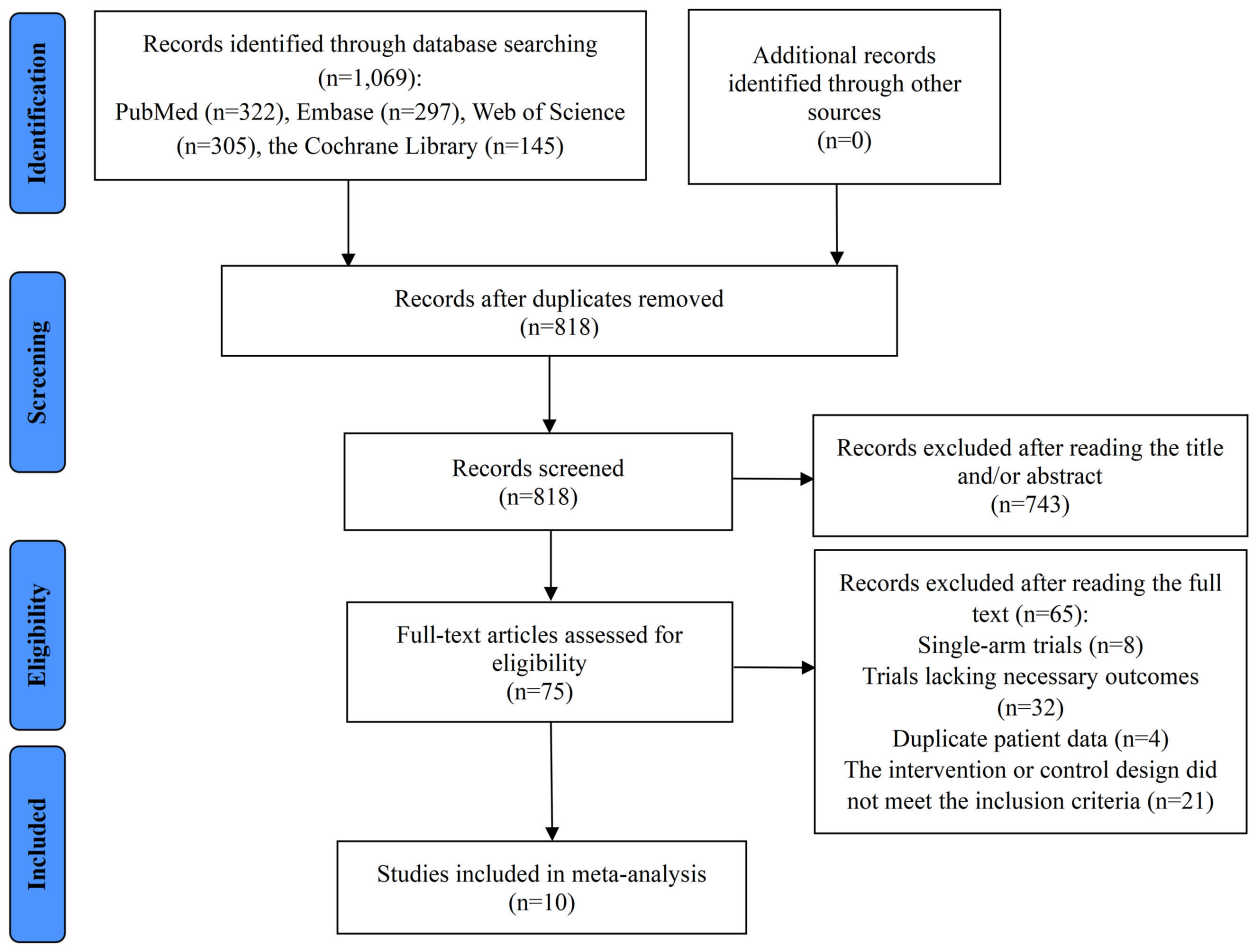
Flow diagram of the process of study selection.

Database searches identified 1,069 records. After duplicate removal, 818 records underwent title-and-abstract screening. Of these, 743 were excluded as irrelevant, leaving 75 for full-text review. Upon further assessment, 65 studies were excluded for various reasons: 8 were single-arm trials, 32 did not report the required outcome data, 4 contained overlapping patient data, and 21 had intervention or control designs that were incompatible with the inclusion criteria. Ultimately, 10 RCTs met the eligibility criteria and were included in the meta-analysis (10, 24–26, 37–42). The study selection process is illustrated in **Figure 1**.

### 3.2 Characteristics and quality assessment of included studies

**Table 1** summarizes the main features of the trials. This meta-analysis incorporated 8 phase II and 2 phase III RCTs, all published in English between 2010 and 2025. Participants were categorized based on GBM status as either newly diagnosed or recurrent cases. Collectively, 1,435 patients were enrolled, with 853 randomized to receive TKI combination therapies and 582 assigned to control arms. According to the primary mechanism of action of the TKIs, the trials were categorized into antiangiogenic (VEGFR-targeted) TKI-based combinations (cediranib, anlotinib, and vandetanib) and non-antiangiogenic (non-VEGFR-targeted) TKI-based combinations (dasatinib, imatinib, gefitinib, afatinib, and neratinib). The therapeutic regimens combined with TKIs were classified into standard CRT (Stupp regimen), which consisted of RT with concurrent TMZ followed by maintenance TMZ, and other non-standard treatment protocols. In the control group, the treatment regimens were identical to those in the intervention group but either replaced TKIs with placebo or omitted TKIs entirely. Of the 10 included trials, 7 were assessed as high quality, while the remaining 3 were rated as low quality due to their open-label design and incomplete reporting of randomization procedures and allocation concealment. Detailed quality assessment results are available in **Supplementary File 2**.

**Table 1:** Main characteristics of the included studies.

Study (year)	Phase	Country	Patient characteristics	Sample size (E/C)	Median (range)/Mean ± SD (E/C, years)	Experimental group	Control group	Outcomes
Batchelor et al. (2013) (24)	III	Multiple countries	Patients aged ≥18 years with recurrent GBM who previously received a TMZ-containing CT regimen and radiotherapy	129/65	54.0 (median)/54.0 (median)	Cediranib (20 mg, orally, once daily) + Lomustine (110 mg/m^2^, once every 6 weeks as oral capsules)	Placebo (orally, once daily) + Lomustine (110 mg/m^2^, once every 6 weeks as oral capsules)	PFS, OS, ORR, SD, PD, and AEs
Galanis et al. (2019) (40)	II	USA	Patients ≥18 years of age with histologically confirmed GBM (including the gliosarcoma variant), an ECOG PS of 0-2, and who had received up to two prior CT regimens (no more than one for recurrent disease)	83/38	58.0 (29.0-79.0)/56.5 (18.0-71.0)	Dasatinib (100 mg, orally, twice daily, from day 1–14 of each 14-day cycle) + Bevacizumab (10 mg/kg, intravenously, on day 1 of each 14-day cycle)	Placebo (orally, twice daily, from day 1–14 of each 14-day cycle) + Bevacizumab (10 mg/kg, intravenously, on day 1 of each 14-day cycle)	PFS, OS, and AEs
Rahman et al. (2023) (42)	II	Multiple countries	Adult patients with newly diagnosed and untreated intracranial GBM or gliosarcoma	81/71	60 (24-78)/59 (24-75)	Neratinib (orally at 240 mg once daily) + TMZ (75 mg/m^2^ orally, once daily) + Radiation therapy (60 Gy in 30 fractions) followed by 6 cycles of adjuvant TMZ 150–200 mg/m^2^ once per day for 5 days in 28-day cycles	TMZ (75 mg/m^2^ orally, once daily) + Radiation therapy (60 Gy in 30 fractions) followed by 6 cycles of adjuvant TMZ 150–200 mg/m^2^ once per day for 5 days in 28-day cycles	PFS, OS, ORR, SD, PD, and AEs
Dresemann et al. (2010) (39)	III	Multiple countries	Adult patients with confirmed recurrent GBM and an ECOG PS of 0–2 who had completed previous treatment comprising surgical resection, irradiation therapy, and first-line CT and who have progressed despite treatment	120/120	52 (26-73)/51 (19-73)	Imatinib (600 mg/d) + Hydroxyurea (1,000 mg/d, 500 mg twice daily)	Hydroxyurea (1,500 mg/d, 500 mg three times daily)	PFS, OS, and AEs
Breen et al. (2025) (25)	II	USA	Patients (age ≥ 18 years) with newly diagnosed histologic GBM with ECOG PS of 0-2	138/66	60.0 (29.0-82.0)/59.5 (29.0-80.0)	Dasatinib (150 mg, orally, once daily) + TMZ (75 mg/m^2^ orally daily) + Radiation therapy (60 Gy in 30 fractions) followed by dasatinib (150 mg) + TMZ (150–200 mg/m^2^ for 5 consecutive days in 28-day cycles)	Placebo (orally, once daily) + TMZ (75 mg/m^2^ orally daily) + Radiation therapy (60 Gy in 30 fractions) followed by placebo + TMZ (150–200 mg/m^2^ for 5 consecutive days in 28-day cycles)	PFS, OS, and AEs
Reardon et al. (2015) (10)	II	North America	Patients (age ≥ 18 years) with histologically confirmed WHO grade 4 malignant glioma at first recurrence after TMZ chemoradiotherapy	39/39	55.4 ± 11.02/56.9 ± 10.62	Afatinib (40 mg/day, orally) + TMZ (75 mg/m^2^, orally, for 21/28 days)	TMZ (75 mg/m^2^, orally, for 21/28 days)	PFS, ORR, SD, PD, and AEs
Brown et al. (2016) (38)	II	UK	Patients (age ≥ 18 years) with histologically confirmed recurrent/progressive GBM, following surgery, cranial radiotherapy and CT with concurrent and adjuvant TMZ	19/19	55.0 (30-71)/61.0 (40-69)	Gefitinib (500 mg, orally, every day) + Cediranib (30 mg, orally, every day)	Placebo (orally, every day) + Cediranib (30 mg, orally, every day)	PFS, OS, ORR, and AEs
Chen et al. (2025) (26)	II	China	Patients (age ≥ 18 years) with newly diagnosed GBM and an ECOG PS of 0-2	77/76	55.4 (median)/55.1 (median)	Anlotinib (10 mg/day, days 1–14 per 21-day cycle) + TMZ-based chemoradiotherapy (54–60 Gy)	Placebo (days 1–14 per 21-day cycle) + TMZ-based chemoradiotherapy (54–60 Gy)	PFS, ORR, and AEs
Batchelor et al. (2023) (37)	II	USA	Patients (age ≥ 18 years) with newly pathologically diagnosed GBM and Karnofsky PS ≥ 70	97/52	61 (27-83)/59 (37-82)	Cediranib (20 mg) + TMZ (75 mg/m^2^/daily) + Radiotherapy (60 Gy in 2 Gy fractions) followed by cediranib (20 mg) + TMZ (150–200 mg/m^2^ for 5 consecutive days in 28-day cycles)	Placebo + TMZ (75 mg/m^2^/daily) + Radiotherapy (60 Gy in 2 Gy fractions) followed by placebo + TMZ (150–200 mg/m^2^ for 5 consecutive days in 28-day cycles)	PFS, OS, and AEs
Lee et al. (2015) (41)	II	USA	Patients (age ≥ 18 years) with histologically confirmed GBM or gliosarcoma who had received no prior CT or radiation	70/36	59 (23-83)/55 (23-73)	Vandetanib (100 mg, orally, daily) + Radiotherapy (daily fractions of 180–200 cGy) + Concurrent TMZ (75 mg/m^2^, orally) + Adjuvant TMZ (150 mg/m^2^/dayvant mg/m^2^/day, orally)	Radiotherapy (daily fractions of 180–200 cGy) + Concurrent TMZ (75 mg/m^2^, orally) + Adjuvant TMZ (150 mg/m^2^/dayvant mg/m^2^/day, orally)	OS, ORR, SD, PD, and AEs

E, Experimental group; C, Control group; SD, standard deviation; GBM, glioblastoma; TMZ, temozolomide; CT, chemotherapy; ECOG, Eastern Cooperative Oncology Group; PS, performance status; TMZ, temozolomide; PFS, progression-free survival; OS, overall survival; ORR, objective response rate; SD, stable disease; PD, progressive disease; AEs, adverse events.

### 3.3 Overall and subgroup analysis of the primary outcomes

#### 3.3.1 PFS

**Figure 2:**
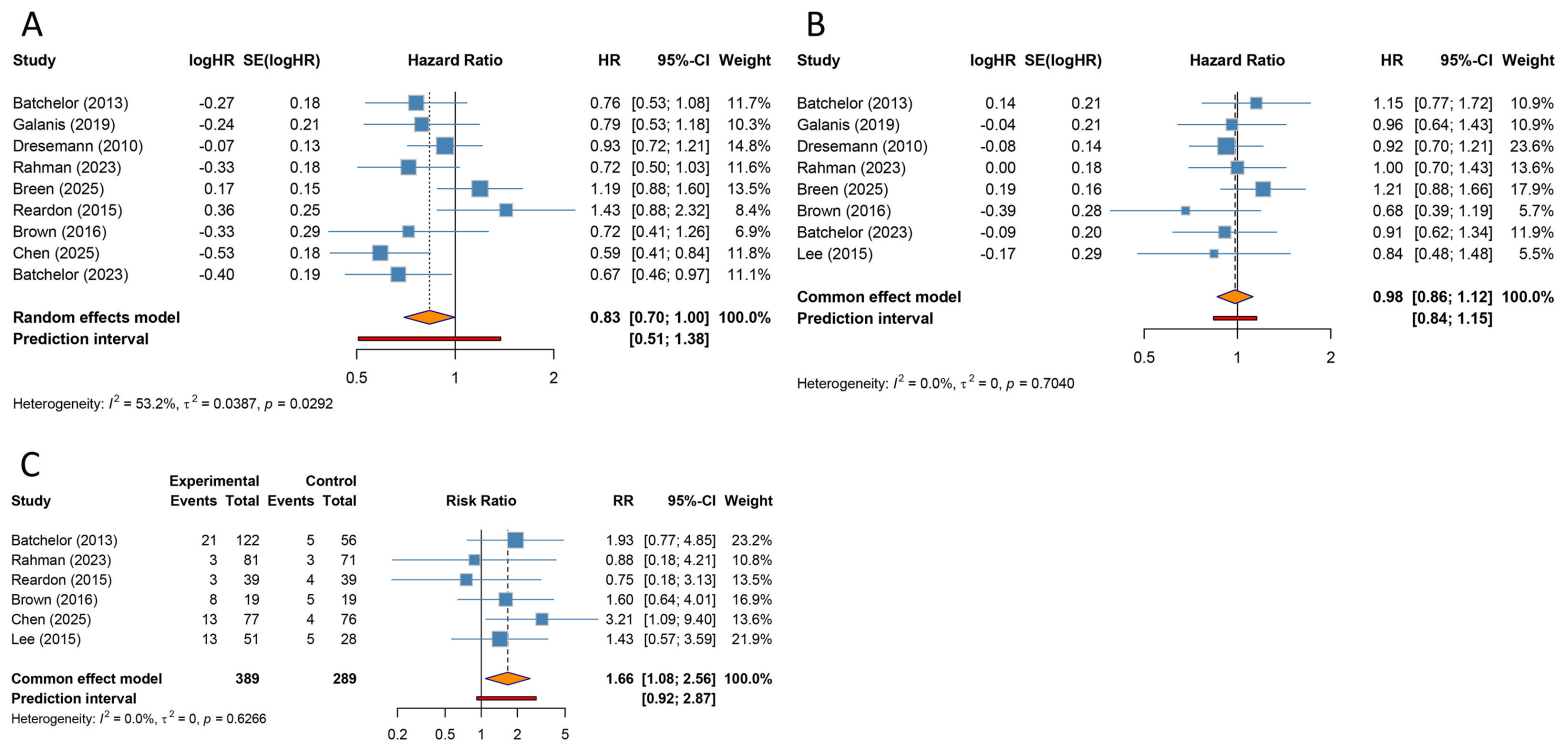
Forest plots of the primary outcomes after tyrosine kinase inhibitor combination therapy for glioblastoma. **(A)** Progression-free survival; **(B)** Overall survival; **(C)** Objective response rate.

PFS outcomes were obtained from 9 included RCTs. Due to substantial heterogeneity among the studies (I^2^ = 53.2%), a random-effects model was employed. The pooled analysis indicated that combination therapies including TKIs significantly improved PFS in patients with GBM compared with control treatments (HR [95% CI] = 0.834 [0.697-0.998], 95% PI: 0.506-1.376) (**Table 2**, **Figure 2A**). However, further subgroup analyses revealed that neither TKIs combined with standard CRT nor TKIs combined with other non-standard treatment regimens produced a statistically significant PFS benefit versus their respective controls (all *p* > 0.05). Likewise, when stratified by disease status (newly diagnosed versus recurrent GBM), the addition of TKIs did not confer a PFS advantage (all *p* > 0.05). In subgroup analyses stratified by TKI class, angiogenesis-targeting TKI-based combinations were associated with a statistically significant improvement in PFS compared with controls (HR [95% CI] = 0.669 [0.544-0.824], 95% PI: 0.424-1.056), whereas non-angiogenesis-targeting TKI-based combinations showed no evidence of a PFS benefit (HR [95% CI] = 0.945 [0.817-1.094], 95% PI: 0.566-1.556) (**Table 2**; **Supplementary Figures 1**, **2**).

**Table 2:** Overall and subgroup analysis of the efficacy outcomes after tyrosine kinase inhibitor combination therapy for glioblastoma.

Outcomes and subgroups	Number of studies	Meta-analysis				Heterogeneity	
		HR/RR	95% CI	*p* value	95% PI	I^2^, Tau^2^	*p* value
PFS							
Overall	9	0.834	0.697-0.998	0.047	0.506-1.376	53.2%, 0.039	0.029
Treatment regimens							
TKIs plus standard CRT vs. Standard CRT alone (or with placebo)	4	0.771	0.556-1.069	0.119	0.271-2.196	72.3%, 0.080	0.013
TKIs plus non-standard therapies vs. Non-standard therapies alone (or with placebo)	5	0.890	0.754-1.052	0.172	0.576-1.376	26.2%, 0.014	0.247
Disease status							
Newly diagnosed GBM	4	0.771	0.556-1.069	0.119	0.271-2.196	72.3%, 0.080	0.013
Recurrent GBM	5	0.890	0.754-1.052	0.172	0.576-1.376	26.2%, 0.014	0.247
TKI class							
Angiogenesis-targeting	3	0.669	0.544-0.824	<0.001	0.424-1.056	0%, 0	0.612
Non-angiogenesis-targeting	6	0.945	0.817-1.094	0.449	0.566-1.556	44.4%, 0.028	0.109
OS							
Overall	8	0.983	0.860-1.122	0.797	0.837-1.154	0%, 0	0.704
Treatment regimens							
TKIs plus standard CRT vs. Standard CRT alone (or with placebo)	4	1.028	0.850-1.243	0.780	0.755-1.399	0%, 0	0.587
TKIs plus non-standard therapies vs. Non-standard therapies alone (or with placebo)	4	0.942	0.782-1.134	0.526	0.696-1.273	0%, 0	0.513
Disease status							
Newly diagnosed GBM	4	1.028	0.850-1.243	0.780	0.755-1.399	0%, 0	0.587
Recurrent GBM	4	0.942	0.782-1.134	0.526	0.696-1.273	0%, 0	0.513
TKI class							
Angiogenesis-targeting	3	0.981	0.764-1.259	0.878	0.567-1.696	0%, 0	0.596
Non-angiogenesis-targeting	5	0.984	0.841-1.151	0.835	0.787-1.228	0%, 0	0.463
ORR							
Overall	6	1.664	1.083-2.558	0.020	0.917-2.868	0%, 0	0.627
Treatment regimens							
TKIs plus standard CRT vs. Standard CRT alone (or with placebo)	3	1.823	0.977-3.402	0.059	0.337-9.081	7.8%, 0.029	0.338
TKIs plus non-standard therapies vs. Non-standard therapies alone (or with placebo)	3	1.527	0.843-2.766	0.162	0.413-5.570	0%, 0	0.547
Disease status							
Newly diagnosed GBM	3	1.823	0.977-3.402	0.059	0.337-9.081	7.8%, 0.029	0.338
Recurrent GBM	3	1.527	0.843-2.766	0.162	0.413-5.570	0%, 0	0.547
TKI class							
Angiogenesis-targeting	3	2.039	1.172-3.547	0.012	0.582-6.740	0%, 0	0.530
Non-angiogenesis-targeting	3	1.132	0.567-2.258	0.726	0.260-5.453	0%, 0	0.617
Components of ORR							
Complete response rate	5	2.435	0.614-9.656	0.206	0.259-22.862	0%, 0	0.999
Partial response rate	5	1.247	0.756-2.059	0.388	0.621-2.618	0%, 0	0.611
Stable disease							
Overall	4	1.011	0.769-1.330	0.936	0.451-2.270	59.2%, 0.045	0.062
Progressive disease							
Overall	4	0.694	0.376-1.279	0.241	0.101-4.754	71.0%, 0.268	0.016

PFS, progression-free survival; TKIs, tyrosine kinase inhibitors; CRT, chemoradiotherapy; GBM, glioblastoma; OS, overall survival; ORR, objective response rate.

#### 3.3.2 OS

8 included studies evaluated OS outcomes. As no significant heterogeneity was observed among the studies (I^2^ = 0%), a fixed-effects model was applied. The overall analysis indicated that TKI-containing combination therapies did not improve OS in patients with GBM compared with controls (HR [95% CI] = 0.983 [0.860-1.122], 95% PI: 0.837-1.154) (**Table 2**, **Figure 2B**). Subgroup analyses based on combination treatment regimens further revealed that TKI-containing combination therapies, whether combined with standard CRT or other non-standard treatment protocols, did not significantly impact OS (all *p* > 0.05). Similarly, no significant effect on OS was observed for TKI-containing combination therapies in either newly diagnosed or recurrent GBM patients (all *p* > 0.05). In subgroup analyses by TKI class, no OS benefit was observed in either category. Angiogenesis-targeting TKI-based combinations did not improve OS compared with controls, and similarly non-angiogenesis-targeting TKI-based combinations showed no significant effect on OS (all *p* > 0.05) (**Table 2**; **Supplementary Figures 3**, **4**).

#### 3.3.3 ORR

We extracted ORR from the 6 included studies. Because there was no significant heterogeneity among studies (I^2^ = 0%), a fixed-effect model was used. The pooled analysis revealed that combination regimens including TKIs were associated with a statistically significant improvement in ORR compared with control treatments (RR [95% CI] = 1.664 [1.083-2.558], 95% PI: 0.917-2.868) (**Table 2**, **Figure 2C**). However, subgroup analyses showed that neither TKIs combined with standard CRT nor TKIs combined with non-standard therapies produced a statistically significant increase in ORR relative to their respective control groups (all *p* > 0.05). Similarly, stratified analyses by disease status (newly diagnosed versus recurrent) did not demonstrate a significant benefit in ORR for TKI-containing combination treatments (all *p* > 0.05). In analyses stratified by TKI class, the improvement in ORR was confined to angiogenesis-targeting TKI-based combinations, which were associated with a higher likelihood of achieving an objective response than control regimens (RR [95% CI] = 2.039 [1.172-3.547], 95% PI: 0.582-6.740). By contrast, non-angiogenesis-targeting TKI-based combinations did not significantly affect ORR (RR [95% CI] = 1.132 [0.567-2.258], 95% PI: 0.260-5.453). Notably, when the components of ORR were examined separately, TKI-containing combinations did not yield significant improvements in either complete response (CR) rate or partial response (PR) rate (all *p* > 0.05) (**Table 2**; **Supplementary Figures 5**-**7**).

### 3.4 Overall and subgroup analysis of the secondary outcomes

#### 3.4.1 SD and PD

**Figure 3:**
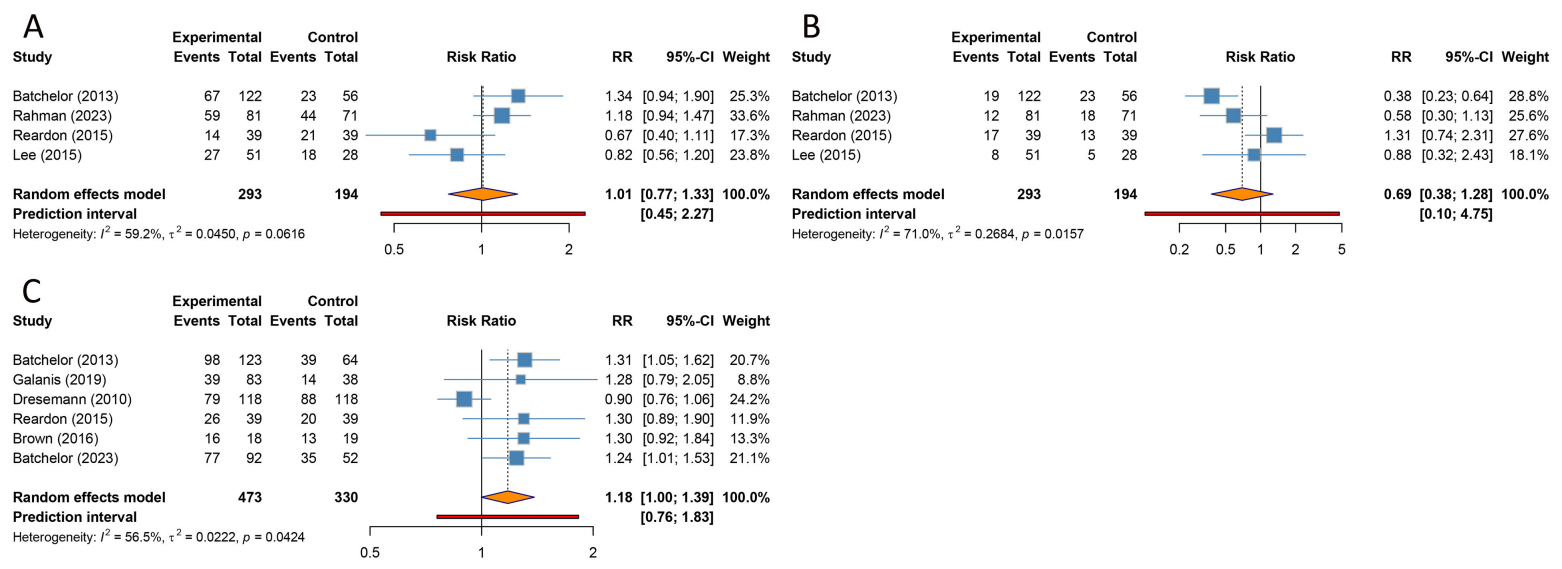
Forest plots of the secondary outcomes after tyrosine kinase inhibitor combination therapy for glioblastoma. **(A)** Stable disease; **(B)** Progressive disease; **(C)** Grade ≥ 3 adverse events.

4 trials provided data on SD and PD outcomes, respectively. Due to substantial heterogeneity among the included studies for both outcomes (SD: I^2^ = 59.2%, PD: I^2^ = 71.0%), a random-effects model was applied. The combined analyses indicated that combination regimens containing TKIs conferred no significant difference in rates of SD (RR [95% CI] = 1.011 [0.769-1.330], 95% PI: 0.451-2.270) or PD (RR [95% CI] = 0.694 [0.376-1.279], 95% PI: 0.101-4.754) compared with control regimens without TKIs in patients with GBM (**Figures 3A, B**). Given the small number of studies available for these endpoints, further subgroup analyses have not yet been conducted (**Table 2**).

#### 3.4.2 Grade ≥ 3 AEs

6 studies focused on grade ≥ 3 AEs following combination therapies. Substantial heterogeneity was observed among the included studies (I^2^ = 56.5%); therefore, a random-effects model was employed. The pooled analysis showed that compared with control regimens, combination treatments that included TKIs were not associated with an increased risk of overall grade ≥ 3 AEs (RR [95% CI] = 1.177 [0.998-1.388], 95% PI: 0.758-1.826) (**Table 3**, **Figure 3C**). This non-significant finding was corroborated in subgroup analysis of TKIs combined with non−standard therapies and in patients with recurrent GBM (all *p* > 0.05). However, subgroup analysis from a single included study suggested an increased risk of grade ≥ 3 AEs in patients receiving TKIs combined with standard CRT and in the subgroup with newly diagnosed GBM (RR [95% CI] = 1.244 [1.008-1.534]). In subgroup analyses by TKI class, angiogenesis-targeting TKI-based combinations were associated with a higher risk of grade ≥ 3 AEs than control regimens (RR [95% CI] = 1.278 [1.098-1.486], 95% PI: 0.481-3.377). By contrast, non-angiogenesis-targeting TKI-based combinations did not show evidence of an increased risk of grade ≥3 AEs (RR [95% CI] = 1.125 [0.885-1.430], 95% PI: 0.564-2.243) (**Table 3**; **Supplementary Figures 8**, **9**).

**Table 3:** Overall and subgroup analysis of the safety outcomes after tyrosine kinase inhibitor combination therapy for glioblastoma.

Outcomes and subgroups	Number of studies	Meta-analysis				Heterogeneity	
		RR	95% CI	*p* value	95% PI	I^2^, Tau^2^	*p* value
Grade ≥ 3 AEs							
Overall	6	1.177	0.998-1.388	0.053	0.758-1.826	56.5%, 0.022	0.042
Treatment regimens							
TKIs plus standard CRT vs. Standard CRT alone (or with placebo)	1	1.244	1.008-1.534	0.042			
TKIs plus non-standard therapies vs. Non-standard therapies alone (or with placebo)	5	1.168	0.950-1.435	0.140	0.659-2.071	61.6%, 0.032	0.034
Disease status							
Newly diagnosed GBM	1	1.244	1.008-1.534	0.042			
Recurrent GBM	5	1.168	0.950-1.435	0.140	0.659-2.071	61.6%, 0.032	0.034
TKI class							
Angiogenesis-targeting	2	1.278	1.098-1.486	0.002	0.481-3.377	0%, 0	0.742
Non-angiogenesis-targeting	4	1.125	0.885-1.430	0.337	0.564-2.243	55.4%, 0.032	0.081
Common AEs of grade ≥ 3							
Hematologic disorders							
Thrombocytopenia	4	1.123	0.484-2.606	0.788	0.091-13.912	65.2%, 0.441	0.035
Lymphopenia	4	1.384	0.830-2.308	0.213	0.337-5.165	16.5%, 0.080	0.309
Neutropenia	3	2.715	1.279-5.763	0.009	0.053-93.614	48.5%, 0.448	0.143
Leukopenia	3	1.190	0.647-2.188	0.577	0.077-19.937	39.3%, 0.227	0.192
WBC decreased	3	1.196	0.552-2.591	0.651	0.203-6.702	0%, 0	0.484
Lymphocyte count decreased	3	0.602	0.444-0.815	0.001	0.252-1.360	8.2%, 0.010	0.336
Hepatic abnormalities							
ALT increased	3	1.261	0.474-3.350	0.642	0.004-278.152	40.1%, 0.942	0.188
Clinical symptomatic AEs							
Fatigue	5	1.936	1.069-3.509	0.029	0.742-3.986	0%, 0	0.786
Hypertension	5	2.430	0.578-10.214	0.225	0.056-105.271	51.5%, 1.306	0.083
Diarrhea	5	3.511	1.041-11.847	0.043	0.587-18.962	0%, 0	0.975
Headache	4	0.536	0.220-1.304	0.169	0.118-2.650	0%, 0	0.464

AEs, adverse events; TKIs, tyrosine kinase inhibitors; CRT, chemoradiotherapy; GBM, glioblastoma; ALT, alanine aminotransferase; WBC, white blood cell.

Regarding specific grade ≥ 3 AEs, we observed that TKI-containing combination therapies increased the risk of neutropenia (RR [95% CI] = 2.715 [1.279-5.763], 95% PI: 0.053-93.614), fatigue (RR [95% CI] = 1.936 [1.069-3.509], 95% PI: 0.742-3.986), and diarrhea (RR [95% CI] = 3.511 [1.041-11.847], 95% PI: 0.587-18.962), while reducing the risk of decreased lymphocyte count (RR [95% CI] = 0.602 [0.444-0.815], 95% PI: 0.252-1.360) (**Table 3**; **Supplementary Figures 10**, **11**).

### 3.5 Trial sequential analysis results

**Figure 4:**
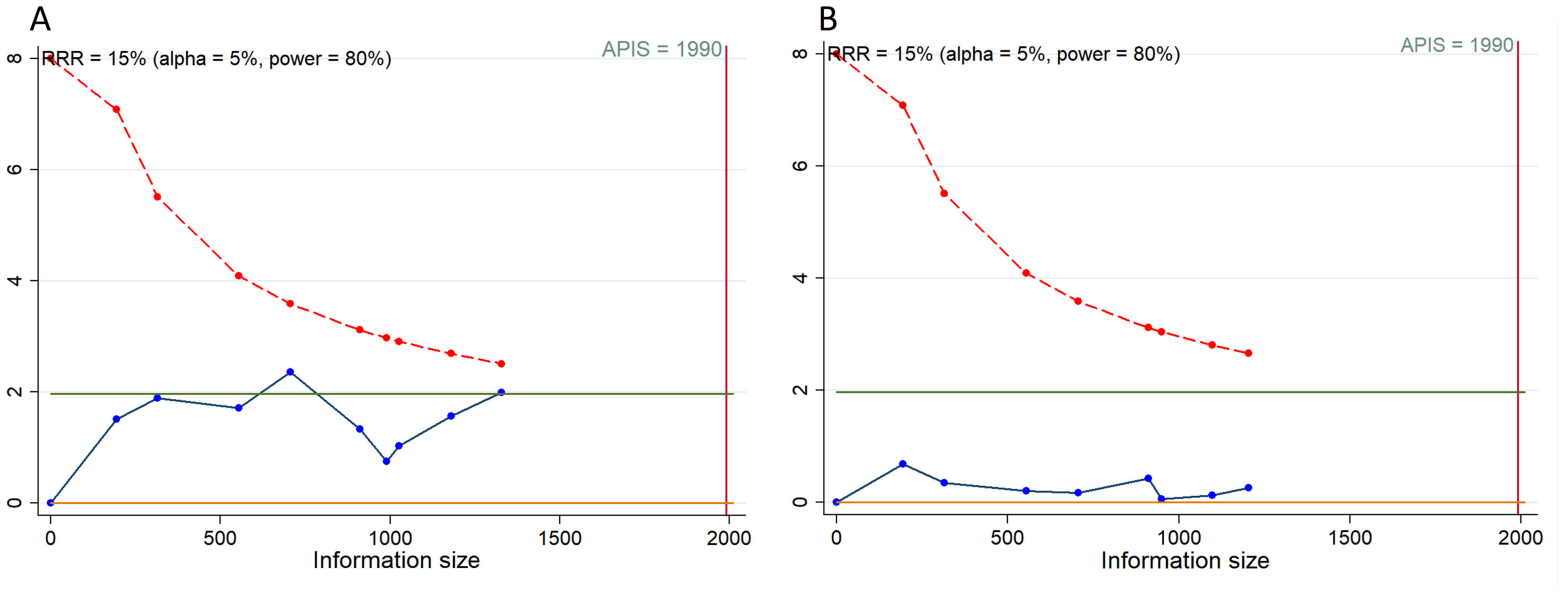
Trial sequential analysis of the survival outcomes after tyrosine kinase inhibitor combination therapy for glioblastoma. **(A)** Progression-free survival; **(B)** Overall survival. Red inward-sloping line to the left represents trial sequential monitoring boundary. Blue line represents evolution of cumulative Z-score. Horizontal green lines represent the conventional boundaries for statistical significance. Heterogeneity-adjusted required information size to demonstrate or reject 15% relative risk (*a priori* estimate) of mortality risk (with alpha of 5% and beta of 20%) is 1,990 patients for PFS and OS (vertical red line). Cumulative Z-curve crossing the trial sequential monitoring boundary or the APIS boundary provides firm evidence of effect.

**Figure 5:**
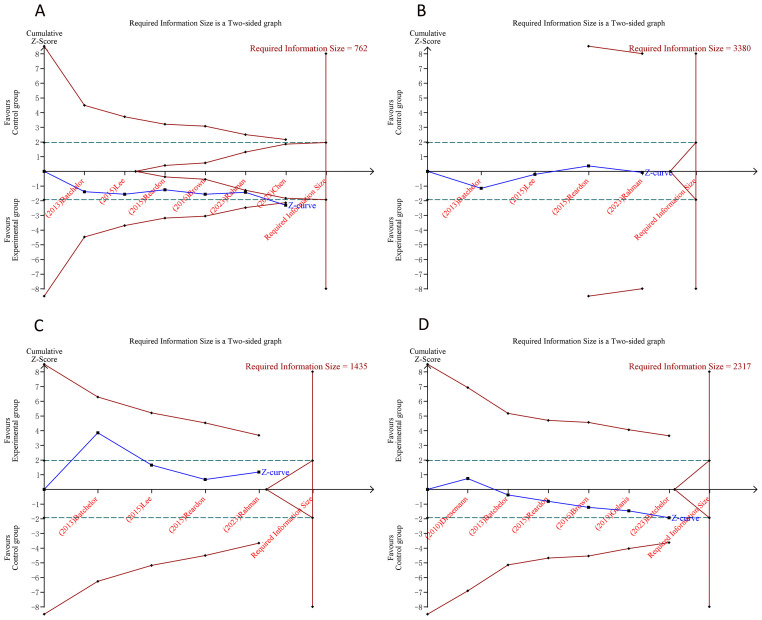
Trial sequential analysis of the dichotomous outcomes after tyrosine kinase inhibitor combination therapy for glioblastoma. **(A)** Objective response rate; **(B)** Stable disease; **(C)** Progressive disease; **(D)** Grade ≥ 3 adverse events. Uppermost and lowermost red curves represent trial sequential monitoring boundary lines for benefit and harm, respectively. Inner red lines represent the futility boundary. Blue line represents evolution of cumulative Z-score. Horizontal green lines represent the conventional boundaries for statistical significance. Cumulative Z-curve crossing the trial sequential monitoring boundary or the RIS boundary provides firm evidence of effect.

Using the pre-specified parameter settings, TSA estimated the APIS for the survival endpoints as 1,990. Similarly, we computed the RIS and constructed trial sequential monitoring boundaries for the dichotomous outcomes using TSA v0.9.5.10 Beta (**Figures 4**, **5**). Notably, the cumulative Z-curve for ORR surpassed the monitoring threshold but did not achieve the RIS, while the Z-curves for all other outcomes failed to cross either the RIS or the monitoring thresholds. These findings indicate that the result for ORR is likely reliable and conclusive, whereas the evidence for the other endpoints remains uncertain and may be susceptible to false-positive interpretation.

### 3.6 Sensitivity analysis and publication bias

Because relatively few studies included in each outcome and to preserve statistical power, sensitivity analyses and publication bias assessments were restricted to outcomes with at least 6 studies included. Sensitivity analyses revealed that the pooled 95% CIs for PFS and ORR were close to 1, indicating that the exclusion of individual studies could substantially influence the pooled estimates. Notably, the removal of the study conducted by Dresemann et al. was found to potentially alter the statistical significance of overall grade ≥ 3 AEs (**Supplementary Figure 12**). Assessments of publication bias, including Begg’s and Egger’s tests alongside visual evaluation of funnel plots, did not identify significant evidence of bias across the analyzed outcomes (**Supplementary Figure 13**).

## 4 Discussion

GBM represents the most prevalent and aggressive primary brain tumor in adults (43). Current standard-of-care involves maximal surgical resection followed by RT and concurrent and adjuvant TMZ chemotherapy (CT). Despite these multimodal interventions, patient survival outcomes remain dismal, with recurrence-driven mortality being a predominant challenge (17). In recent years, targeted therapies have emerged as a promising avenue, aiming to disrupt cancer cell survival by inhibiting key signaling pathways, targeting specific oncogenic proteins, or enabling precise drug delivery to tumor cells while mitigating systemic toxicity (44). Among these, TKIs have revolutionized cancer treatment over the past two decades, with more than 40 molecules approved by the U.S. Food and Drug Administration (FDA) for various malignancies (45). To evaluate their therapeutic potential in GBM, we performed a meta-analysis of 10 RCTs that compared combination regimens incorporating TKIs to comparable regimens without them. The pooled findings revealed that TKI-containing combination therapies significantly improved PFS and ORR in GBM patients. However, no notable benefits were observed in OS or rates of SD and PD. Moreover, the addition of TKIs did not increase the overall risk of grade ≥ 3 AEs.

TKIs target receptor and non-RTKs by several distinct mechanisms, including ATP-competitive blockade of the catalytic site, allosteric modulation, prevention of ligand engagement with RTKs, disruption of kinase-protein interactions, and promotion of kinase destabilization (46). By attenuating oncogenic tyrosine kinase signaling, these agents can suppress tumor cell proliferation and impair the angiogenic programmes that generate and maintain the vascular supply of tumours (47, 48). Preclinical studies have demonstrated the potential of TKIs in treating GBM and brain metastases. For example, imatinib has been shown to enhance the sensitivity of U87 human GBM cells to radiation therapy and amplify the tumor-reducing effects of fractionated RT (49). However, the efficacy of TKI monotherapy in adult GBM patients has been limited in clinical settings. In a phase II study, Muhic et al. administered nintedanib 200 mg twice daily to patients with recurrent GBM after standard first-line therapy and observed only minimal, non-significant anti-tumor activity (50). Similarly, Sharma et al. reported that dovitinib failed to produce a meaningful improvement in PFS among recurrent GBM patients, irrespective of prior exposure to anti-angiogenic therapy (47). These clinical results indicate that, although biologically rational, TKI monotherapy has yet to demonstrate robust efficacy in adult GBM.

Brain tumors function as complex ecosystems characterized by intricate, interconnected networks, which often render single-agent therapies ineffective. In GBM, tumor heterogeneity is further exacerbated by the simultaneous activation of diverse molecular pathways (51). Compounding this challenge, the limited permeability of most TKIs across the BBB significantly restricts their therapeutic efficacy in brain malignancies (52). Consequently, the integration of TKIs into combination treatment strategies has emerged as a potentially more effective approach, particularly in light of the historical shortcomings of monotherapies. For example, the phase II NRG/RTOG 0837 trial reported a longer 6-month PFS when cediranib was added to radiation plus TMZ compared with placebo plus radiation and TMZ (37), and Brown et al. observed a suggestion of better responses and survival in recurrent GBM when gefitinib was combined with cediranib (38). Our pooled analysis further corroborates the therapeutic potential of TKI-based combination regimens, indicating that adding TKIs to various treatment regimens (including the Stupp regimen and other non−standard therapies) significantly improves PFS and ORR in patients with GBM. Nevertheless, subgroup analyses revealed that these improvements in PFS and ORR did not reach statistical significance within the subgroups receiving TKIs combined with the Stupp regimen or with other non−standard therapies, nor when stratified by disease status (newly diagnosed versus recurrent GBM). Several considerations may explain a favorable overall effect estimate alongside non-concordant subgroup results. First, PFS and ORR are more susceptible than OS to assessment-related bias in GBM, because treatment-related effects (including pseudo-progression and changes in BBB permeability) can confound imaging interpretation (53). Second, limited central nervous system exposure for many TKIs, imposed by the BBB and the blood-tumor barrier (54), may restrict the magnitude and durability of true antitumor activity, resulting in only transient disease control. Notably, our subgroup analysis by TKI class suggests that the PFS and ORR benefits were confined to angiogenesis-targeting TKIs, whereas non-anti-angiogenic TKIs showed no clear benefit. These findings are biologically plausible. Anti-angiogenic strategies can induce radiographic responses and delay progression by reducing vascular permeability and contrast enhancement (55), which may translate into PFS/ORR improvements without necessarily altering the underlying infiltrative tumor biology or extending survival. Overall, differences across trials in disease setting, treatment backbones, steroid use and imaging schedules, and-critically-drug class and BBB penetration, may differentially influence radiographic response and time-to-event endpoints. Therefore, these findings should be interpreted with caution. Further clarification will require well-designed trials and individual patient data meta-analyses.

Our study also found that combination regimens including TKIs had no significant effect on OS, rates of SD and PD in patients with GBM. Across the included trials, comparisons of TKI-containing combination therapy versus control showed no statistically significant improvement in OS, as all reported 95% CIs for HRs included 1 (24, 25, 37–42). Likewise, no notable differences were observed in SD or PD rates between the experimental and control cohorts in any of the trials analyzed (10, 24, 41, 42). Consequently, the absence of statistical significance in the pooled OS, SD, and PD outcomes appears consistent with the available evidence. In GBM, whether an improvement in PFS is clinically meaningful when OS does not increase is still debated. In our meta-analysis, regimens combining TKIs showed only a small PFS gain; however, the estimate sat near the threshold of significance, varied notably across studies, and the prediction interval included 1. These results suggest that a clinically relevant benefit is unlikely to be consistent across settings. While delaying radiographic or clinical progression could be valuable by postponing neurological decline, reducing symptom burden, and potentially limiting corticosteroid exposure (56), such benefits may not translate into prolonged survival. Several factors might explain the observed dissociation between PFS and OS. Progression assessment based on imaging is susceptible to misclassification in GBM, particularly in the context of treatment-related phenomena such as pseudoprogression after CRT; the Response Assessment in Neuro-Oncology (RANO) criteria were introduced to address these limitations (53), yet residual inter-study variability persists. Moreover, restricted drug delivery to the central nervous system imposed by the BBB, together with pronounced intratumoural heterogeneity (54), may limit the magnitude and durability of TKI activity, resulting in transient radiographic stabilization without altering the disease’s fatal course. Additionally, the absence of an OS benefit and the substantial heterogeneity in PFS indicate that TKI-containing combinations provide, at most, a modest therapeutic effect in GBM. Limited central nervous system exposure imposed by the BBB and the variable permeability of the blood-tumor barrier are likely to reduce intratumoral drug concentrations, particularly for many small-molecule kinase inhibitors (57). Moreover, GBM is characterized by marked spatial and temporal intratumoural heterogeneity and adaptive resistance (58), which can attenuate the impact of single-pathway inhibition and result in transient radiographic disease control without durable survival benefit. Collectively, these factors may constrain clinical efficacy. Future trials should prioritize biomarker-driven eligibility criteria and, where feasible, mandate pharmacokinetic and pharmacodynamic assessments in tumor tissue and/or cerebrospinal fluid. OS should remain the primary endpoint, with patient-reported outcomes incorporated to capture clinically meaningful benefit beyond imaging-based measures.

Notably, the aggregated safety data showed that TKI-containing combination regimens did not increase the overall risk of grade ≥ 3 AEs. The most frequently reported grade ≥ 3 AEs were hematologic disorders, consistent with a recent meta-analysis (59). These included conditions such as thrombocytopenia, lymphopenia, neutropenia, leukopenia, decreased white blood cell count (WBC), and decreased lymphocyte count. Other commonly reported grade ≥ 3 AEs included alanine aminotransferase (ALT) elevation, fatigue, hypertension, diarrhea, and headache. When compared to control treatments, TKI-containing combinations were associated with higher risks of grade ≥ 3 neutropenia, fatigue, and diarrhea, but with a lower incidence of grade ≥ 3 lymphocyte count decreased. These results underscore the importance of vigilant monitoring of blood parameters and clinical symptoms in patients receiving TKI-containing regimens. Early identification and timely management of AEs, tailored to their severity, are critical to optimizing patient safety and treatment outcomes when these therapies are integrated into existing treatment protocols.

Several limitations remain in this meta-analysis. First, differences in trial design, GBM disease status, patient sample size and characteristics, the specific TKIs used, and the combination therapy regimens across studies may have contributed to the substantial heterogeneity observed in certain outcomes. Second, due to the limited information available from the included studies, subgroup analyses based on molecular biomarkers (e.g., MGMT promoter methylation, IDH status, and key RTK pathway alterations such as EGFR), patient age, sex, and race could not be conducted, warranting further investigation in future research. Additionally, the 10 trials included in this analysis evaluated 8 different small-molecule TKI agents. As more evidence becomes available, it will be critical to prioritize the evaluation of individual agents to better define their therapeutic efficacy and safety profiles in GBM. Third, for certain studies that did not directly report PFS or OS outcomes (10, 39, 41), HRs and corresponding 95% CIs were estimated using the Tierney et al. method. While this approach is commonly employed in meta-analyses, such indirect estimations may have introduced some imprecision into the pooled results. Fourth, TSA indicated that additional studies with larger sample sizes are required to further validate and support the pooled estimates for outcomes other than ORR reported in our study. Fifth, control regimens varied across trials, which may have increased heterogeneity and limited comparability. More RCTs are needed to enable a network meta-analysis that can more comprehensively compare the efficacy and safety across the different treatment strategies.

## 5 Conclusion

In conclusion, our study suggests that combination therapies incorporating TKIs represent a potentially modest treatment strategy for GBM, primarily reflected by improved PFS and higher ORR; however, these benefits did not translate into an improvement in OS in the available RCT evidence, and therefore the clinical impact should be interpreted cautiously. With respect to safety, it is crucial for clinicians to implement proactive and thorough monitoring for hematological adverse effects and other clinical symptoms when incorporating TKIs into treatment regimens, ensuring timely intervention when necessary. Future research should focus on assessing the therapeutic impact and safety profiles of specific TKI agents and exploring the effectiveness of these combination strategies within GBM subtypes defined by molecular biomarkers, with OS and patient-centered outcomes as key endpoints.

## Data Availability

The original contributions presented in the study are included in the article/**Supplementary Material**. Further inquiries can be directed to the corresponding author.
