# Supplementary material for: Efficacy and safety of tyrosine kinase inhibitor combination therapy for glioblastoma: a meta-analysis with trial sequential analysis of randomized controlled trials
Source: Front Oncol. 2026 Apr 20;16:1796708. doi: 10.3389/fonc.2026.1796708 (PMC13135965; doi:10.3389/fonc.2026.1796708)
Supplement: Supplementary file 1 [file DataSheet1.docx]

| **Pubmed: 322** |
| --- |
| #1 ("Glioblastoma"[Mesh] OR glioblastoma[tiab] OR "glioblastoma multiforme"[tiab] OR GBM[tiab] OR "high-grade glioma"[tiab] OR "high grade glioma"[tiab] OR "grade IV glioma"[tiab] OR "malignant glioma"[tiab] OR "astrocytoma grade IV"[tiab]) |
| #2 ("Protein-Tyrosine Kinases"[Mesh] OR "protein tyrosine kinase inhibitor"[tiab] OR "tyrosine kinase inhibitor"[tiab] OR "kinase inhibitor"[tiab] OR TKI[tiab] OR erlotinib[tiab] OR gefitinib[tiab] OR lapatinib[tiab] OR afatinib[tiab] OR dacomitinib[tiab] OR osimertinib[tiab] OR sorafenib[tiab] OR sunitinib[tiab] OR pazopanib[tiab] OR cediranib[tiab] OR axitinib[tiab] OR regorafenib[tiab] OR dasatinib[tiab] OR imatinib[tiab] OR nilotinib[tiab] OR vandetanib[tiab] OR ponatinib[tiab] OR bosutinib[tiab] OR vatalanib[tiab] OR neratinib[tiab]) |
| #3 (randomized controlled trial[pt] OR controlled clinical trial[pt] OR randomized[tiab] OR randomised[tiab] OR randomly[tiab] OR random*[tiab] OR placebo[tiab] OR "double-blind"[tiab] OR "double blind"[tiab] OR "single-blind"[tiab] OR "single blind"[tiab] OR clinical trial[pt]) |
| #4 #1 AND #2 AND #3 |
| **Embase: 297** |
| #1 'glioblastoma'/exp OR glioblastoma:ti,ab OR 'glioblastoma multiforme':ti,ab |
| #2 'tyrosine kinase inhibitor'/exp OR 'tyrosine kinase inhibitor':ti,ab OR 'tyrosine kinase inhibitors':ti,ab OR erlotinib:ti,ab OR gefitinib:ti,ab OR lapatinib:ti,ab OR afatinib:ti,ab OR dacomitinib:ti,ab OR osimertinib:ti,ab OR sorafenib:ti,ab OR sunitinib:ti,ab OR pazopanib:ti,ab OR cediranib:ti,ab OR axitinib:ti,ab OR regorafenib:ti,ab OR dasatinib:ti,ab OR imatinib:ti,ab OR nilotinib:ti,ab OR vandetanib:ti,ab OR ponatinib:ti,ab OR bosutinib:ti,ab OR vatalanib:ti,ab OR neratinib:ti,ab |
| #3 combination:ti,ab OR 'combination therapy':ti,ab OR combined:ti,ab OR concomitant:ti,ab OR concurrent:ti,ab OR plus:ti,ab OR 'combination treatment':ti,ab OR 'combination regimen':ti,ab OR 'combined therapy':ti,ab |
| #4 'randomized controlled trial'/de OR 'randomized clinical trial' OR randomized:ti,ab OR randomised:ti,ab OR randomly:ti,ab OR placebo:ti,ab |
| #5 #1 AND #2 AND #3 AND #4 |
| **Web of Science: 305** |
| #1 TS=(glioblastoma OR "glioblastoma multiforme" OR GBM OR "high-grade glioma" OR "high grade glioma" OR "grade IV glioma" OR "malignant glioma" OR "astrocytoma grade IV") |
| #2 TS=("tyrosine kinase inhibitor" OR "tyrosine kinase inhibitors" OR "protein tyrosine kinase" OR "kinase inhibitor" OR TKI OR erlotinib OR gefitinib OR lapatinib OR afatinib OR dacomitinib OR osimertinib OR sorafenib OR sunitinib OR pazopanib OR cediranib OR axitinib OR regorafenib OR dasatinib OR imatinib OR nilotinib OR vandetanib OR ponatinib OR bosutinib OR vatalanib OR neratinib) |
| #3 TS=(random* OR randomized OR randomised OR randomly OR placebo OR "double blind" OR "double-blind" OR "single blind" OR "clinical trial") |
| #4 #1 AND #2 AND #3 |
| **The Cochrane Library: 145** |
| #1 ([mh "Glioblastoma"] OR glioblastoma:ti,ab,kw OR "glioblastoma multiforme":ti,ab,kw OR GBM:ti,ab,kw OR "high-grade glioma":ti,ab,kw OR "high grade glioma":ti,ab,kw OR "grade IV glioma":ti,ab,kw OR "malignant glioma":ti,ab,kw) |
| #2 ([mh "Protein-Tyrosine Kinase Inhibitors"] OR "tyrosine kinase inhibitor":ti,ab,kw OR "tyrosine kinase inhibitors":ti,ab,kw OR "kinase inhibitor":ti,ab,kw OR TKI:ti,ab,kw OR erlotinib:ti,ab,kw OR gefitinib:ti,ab,kw OR lapatinib:ti,ab,kw OR afatinib:ti,ab,kw OR dacomitinib:ti,ab,kw OR osimertinib:ti,ab,kw OR sorafenib:ti,ab,kw OR sunitinib:ti,ab,kw OR pazopanib:ti,ab,kw OR cediranib:ti,ab,kw OR axitinib:ti,ab,kw OR regorafenib:ti,ab,kw OR dasatinib:ti,ab,kw OR imatinib:ti,ab,kw OR nilotinib:ti,ab,kw OR vandetanib:ti,ab,kw OR ponatinib:ti,ab,kw OR bosutinib:ti,ab,kw OR vatalanib:ti,ab,kw OR neratinib:ti,ab,kw) |
| #3 (random*:ti,ab,kw OR randomized:ti,ab,kw OR randomised:ti,ab,kw OR randomly:ti,ab,kw OR "randomized controlled trial":ti,ab,kw OR "randomised controlled trial":ti,ab,kw OR "clinical trial":ti,ab,kw OR placebo:ti,ab,kw OR "double blind":ti,ab,kw) |
| #4 #1 AND #2 AND #3 |
