# Supplementary material for: Efficacy and safety of tyrosine kinase inhibitor combination therapy for glioblastoma: a meta-analysis with trial sequential analysis of randomized controlled trials
Source: Front Oncol. 2026 Apr 20;16:1796708. doi: 10.3389/fonc.2026.1796708 (PMC13135965; doi:10.3389/fonc.2026.1796708)

**FIGURE S1** Subgroup analysis of progression-free survival based on treatment regimens ([A] tyrosine kinase inhibitors [TKIs] plus standard chemoradiotherapy [CRT] vs. Standard CRT alone [or with placebo]; [B] TKIs plus non-standard therapies vs. Non-standard therapies alone [or with placebo]) and disease status ([C] Newly diagnosed glioblastoma [GBM]; [D] Recurrent GBM).


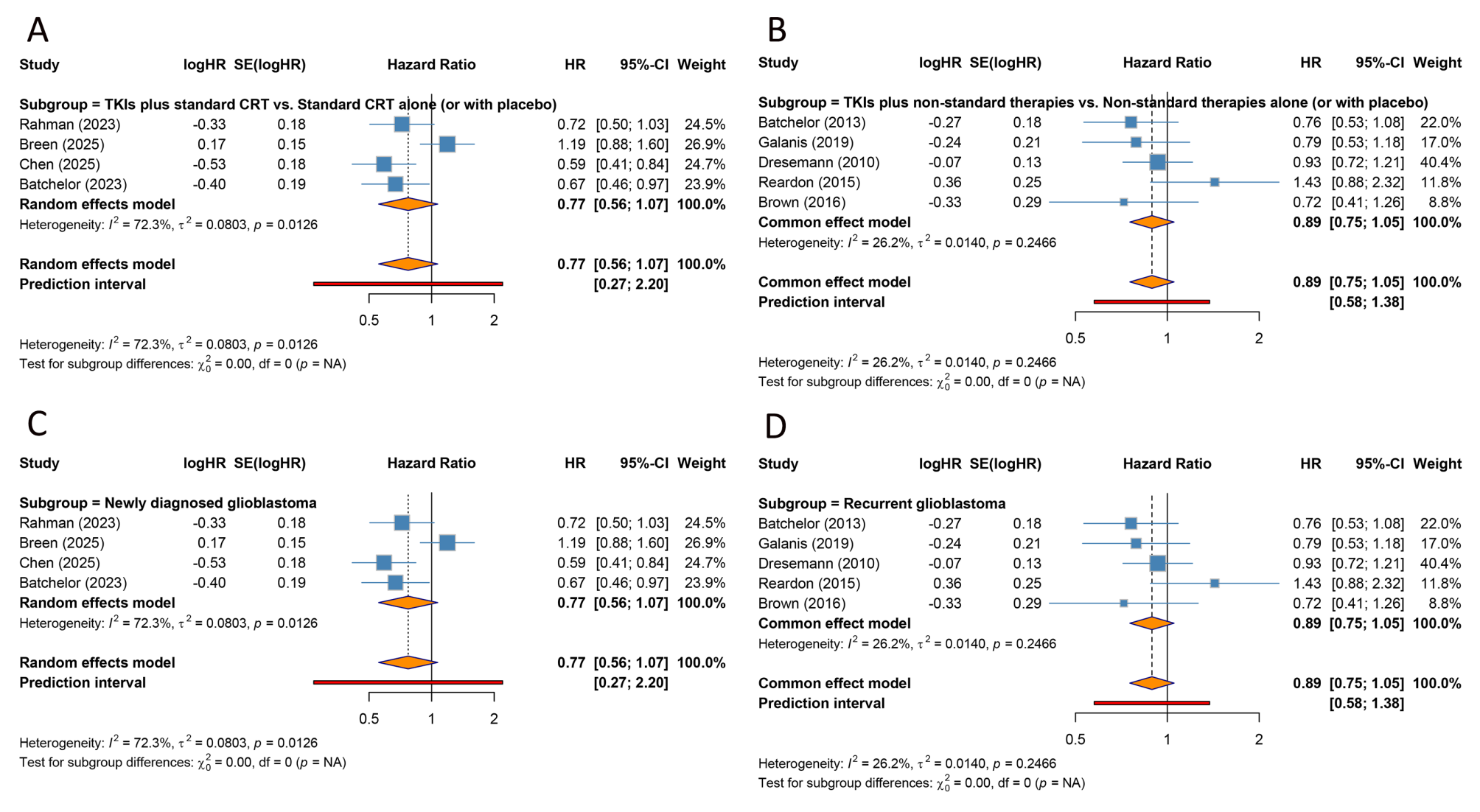


**FIGURE S2** Subgroup analysis of progression-free survival based on tyrosine kinase inhibitor class. (A) Angiogenesis-targeting; (B) Non-angiogenesis-targeting.


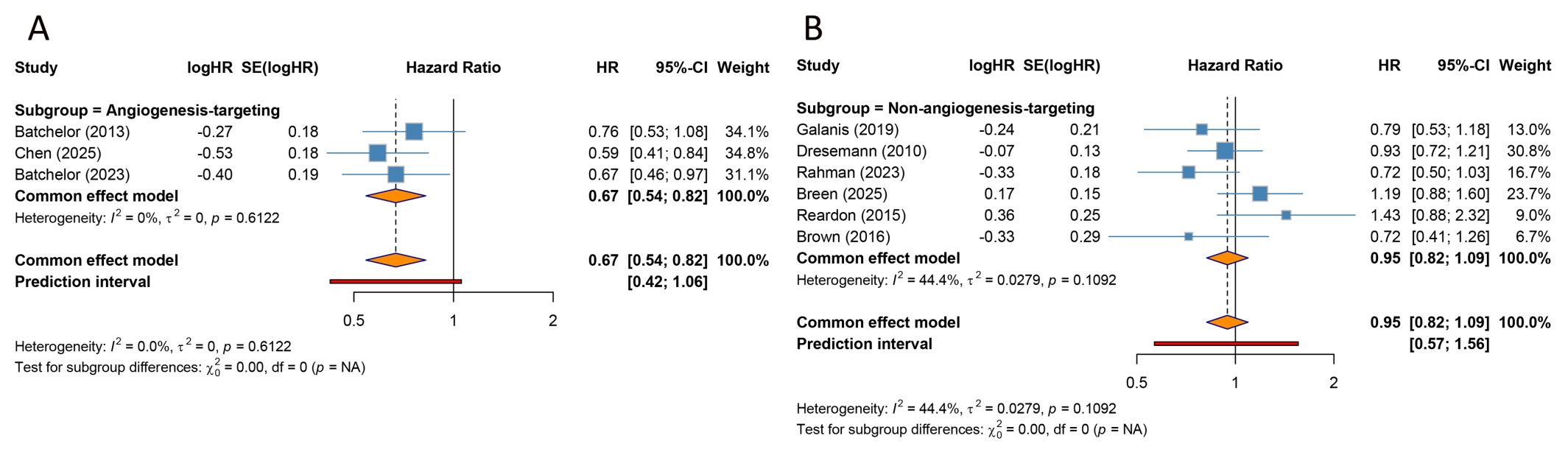


**FIGURE S3** Subgroup analysis of overall survival based on treatment regimens ([A] tyrosine kinase inhibitors [TKIs] plus standard chemoradiotherapy [CRT] vs. Standard CRT alone [or with placebo]; [B] TKIs plus non-standard therapies vs. Non-standard therapies alone [or with placebo]) and disease status ([C] Newly diagnosed glioblastoma [GBM]; [D] Recurrent GBM).


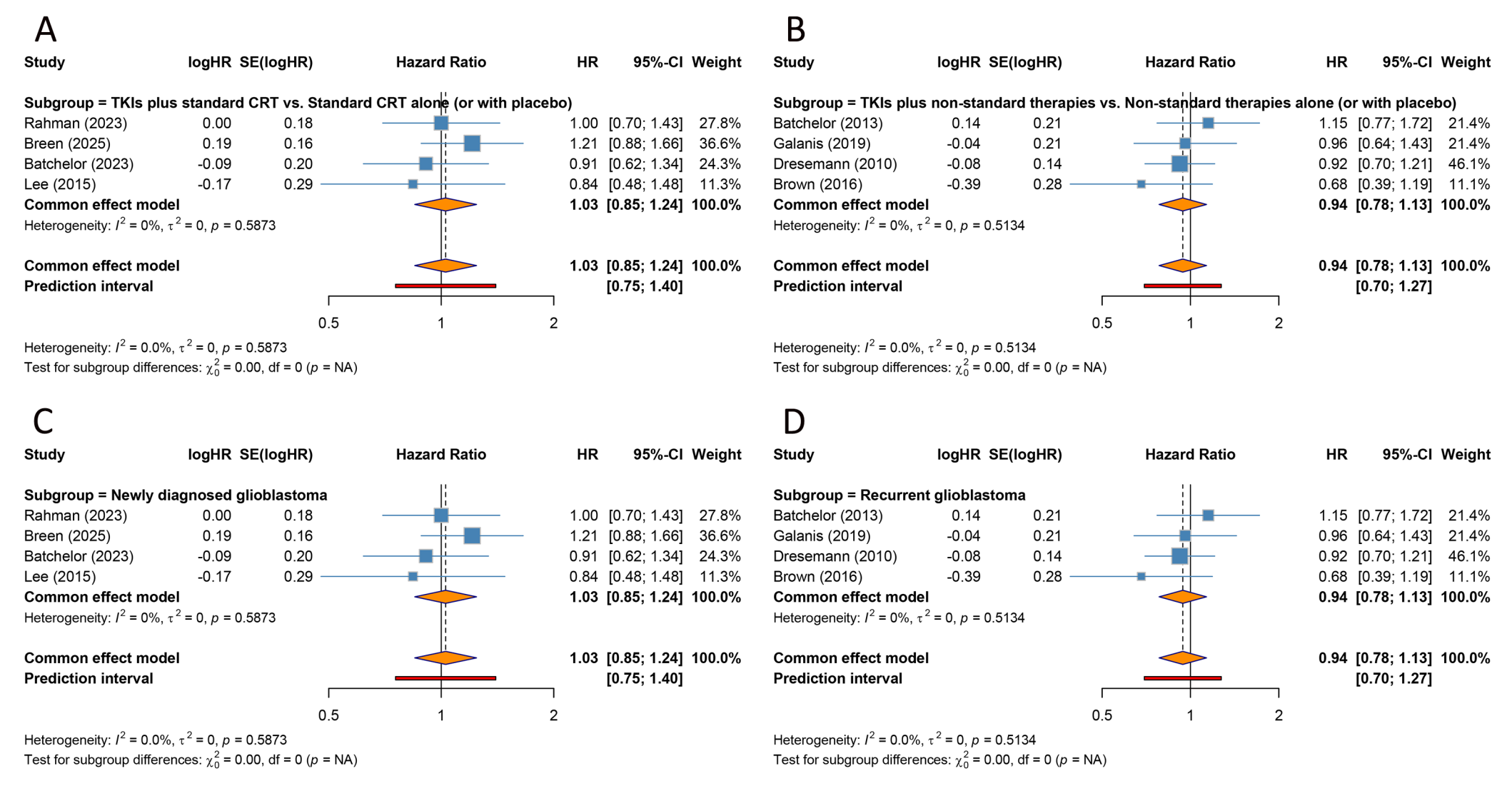


**FIGURE S4** Subgroup analysis of overall survival based on tyrosine kinase inhibitor class. (A) Angiogenesis-targeting; (B) Non-angiogenesis-targeting.


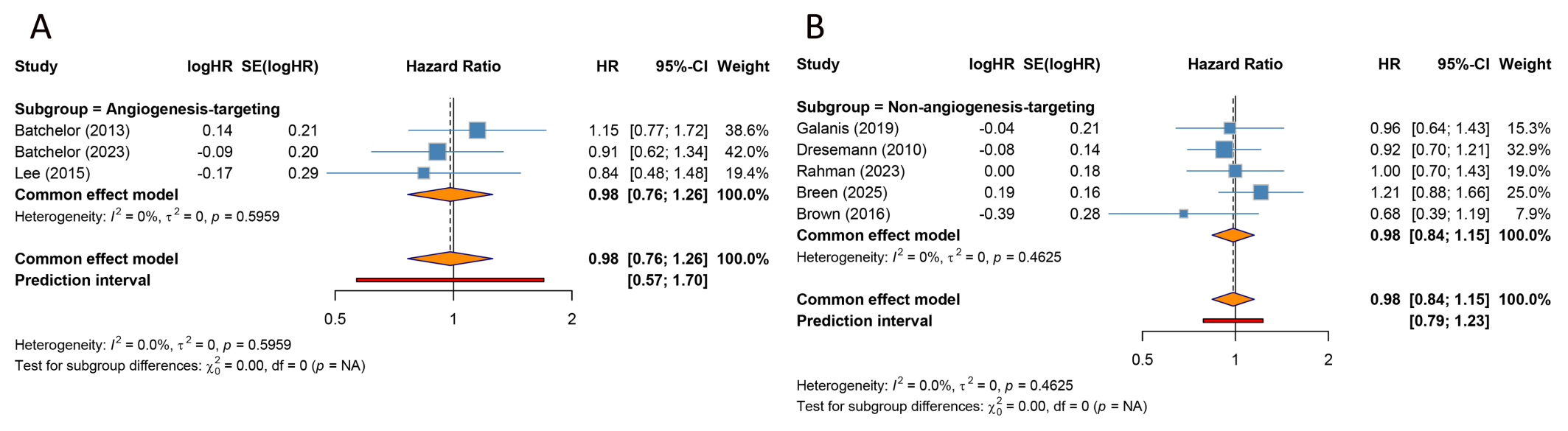


**FIGURE S5** Subgroup analysis of objective response rate (ORR) based on treatment regimens ([A] tyrosine kinase inhibitors [TKIs] plus standard chemoradiotherapy [CRT] vs. Standard CRT alone [or with placebo]; [B] TKIs plus non-standard therapies vs. Non-standard therapies alone [or with placebo]) and disease status ([C] Newly diagnosed glioblastoma [GBM]; [D] Recurrent GBM).


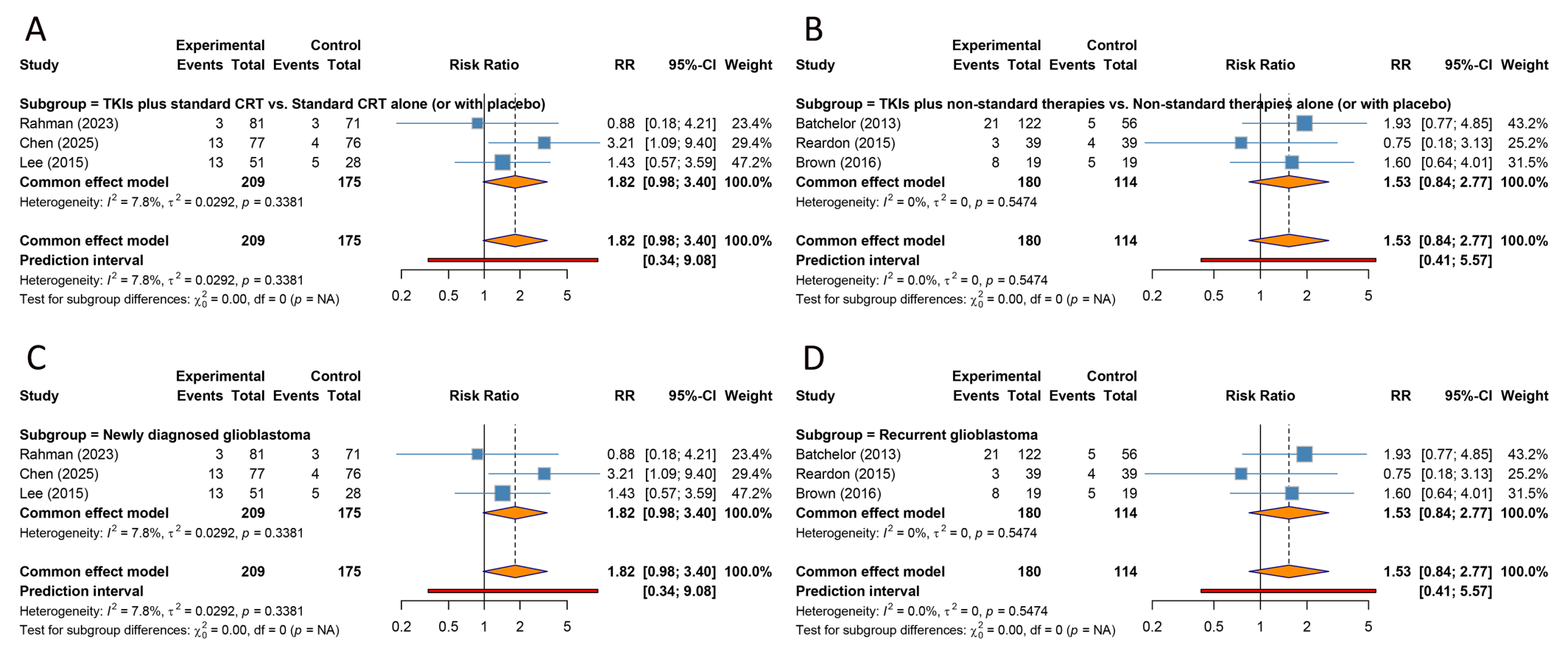


**FIGURE S6** Subgroup analysis of objective response rate based on tyrosine kinase inhibitor class. (A) Angiogenesis-targeting; (B) Non-angiogenesis-targeting.


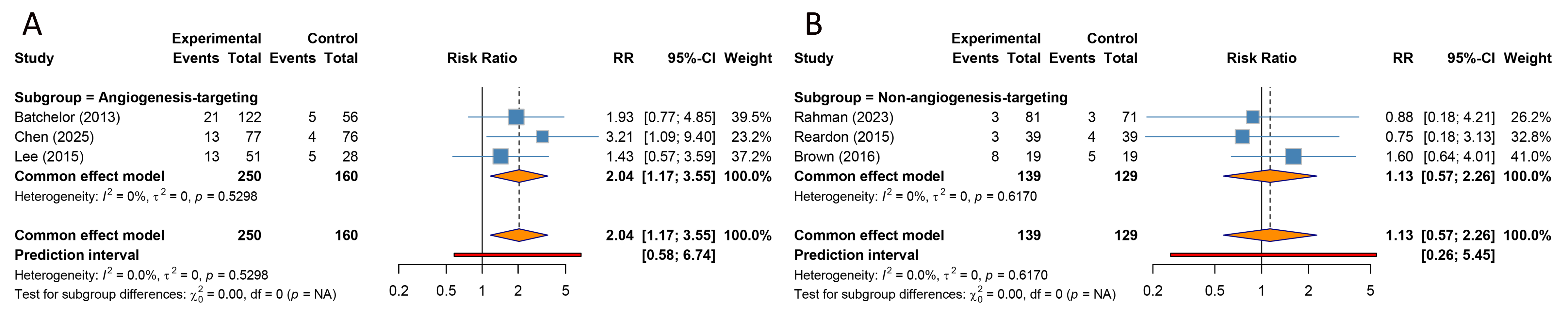


**FIGURE S7** Subgroup analysis of objective response rate (ORR) based on components of ORR ([A] Complete response rate; [B] Partial response rate).


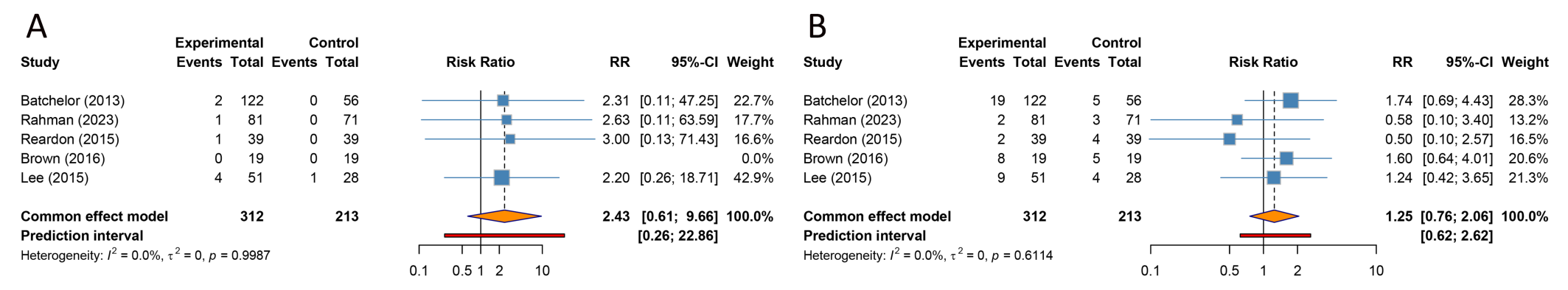


**FIGURE S8** Subgroup analysis of grade ≥ 3 adverse events (AEs) based on treatment regimens ([A] tyrosine kinase inhibitors [TKIs] plus standard chemoradiotherapy [CRT] vs. Standard CRT alone [or with placebo]; [B] TKIs plus non-standard therapies vs. Non-standard therapies alone [or with placebo]) and disease status ([C] Newly diagnosed glioblastoma [GBM]; [D] Recurrent GBM).


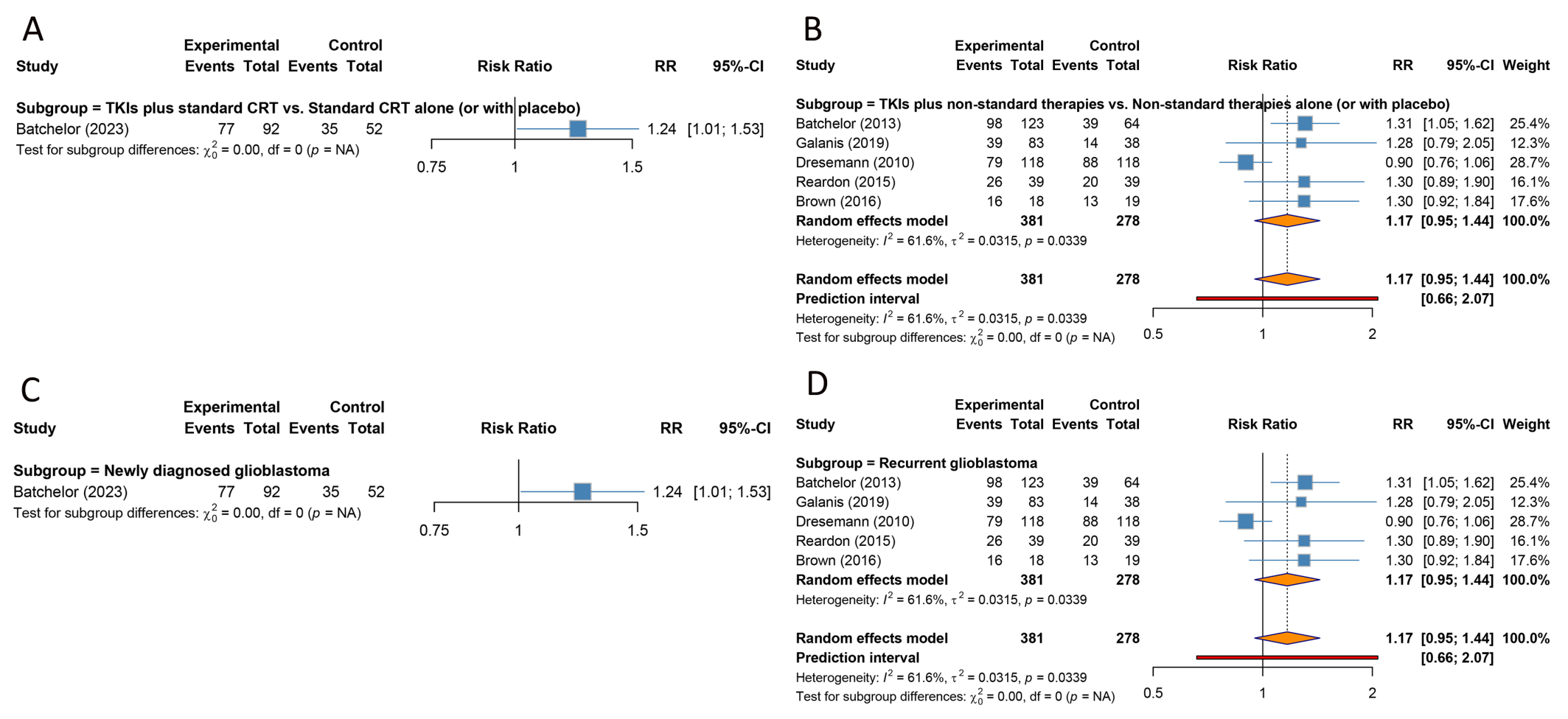


**FIGURE S9** Subgroup analysis of grade ≥ 3 adverse events based on tyrosine kinase inhibitor class. (A) Angiogenesis-targeting; (B) Non-angiogenesis-targeting.


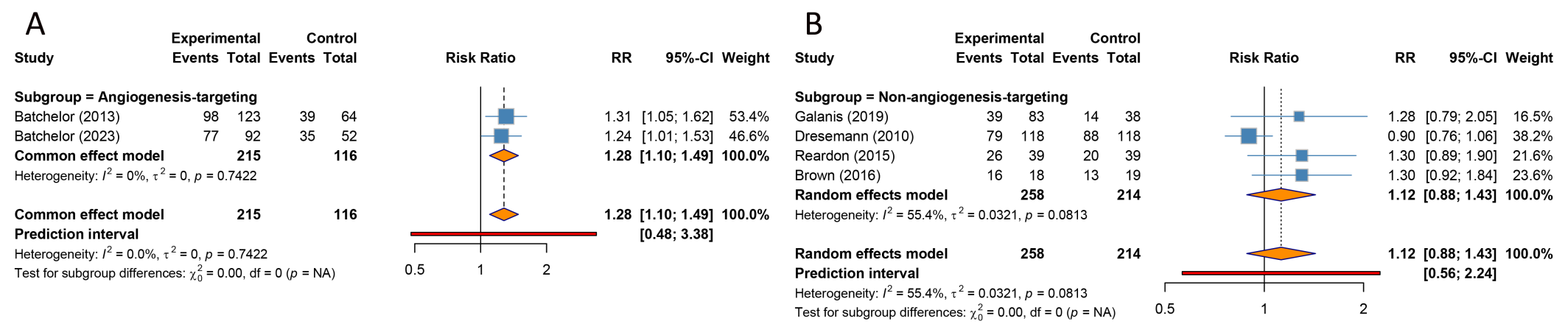


**FIGURE S10** Forest plots of common grade ≥ 3 adverse events (hematologic disorders). (A) Thrombocytopenia; (B) Lymphopenia; (C) Neutropenia; (D) Leukopenia; (E) white blood cell decreased; (F) Lymphocyte count decreased.


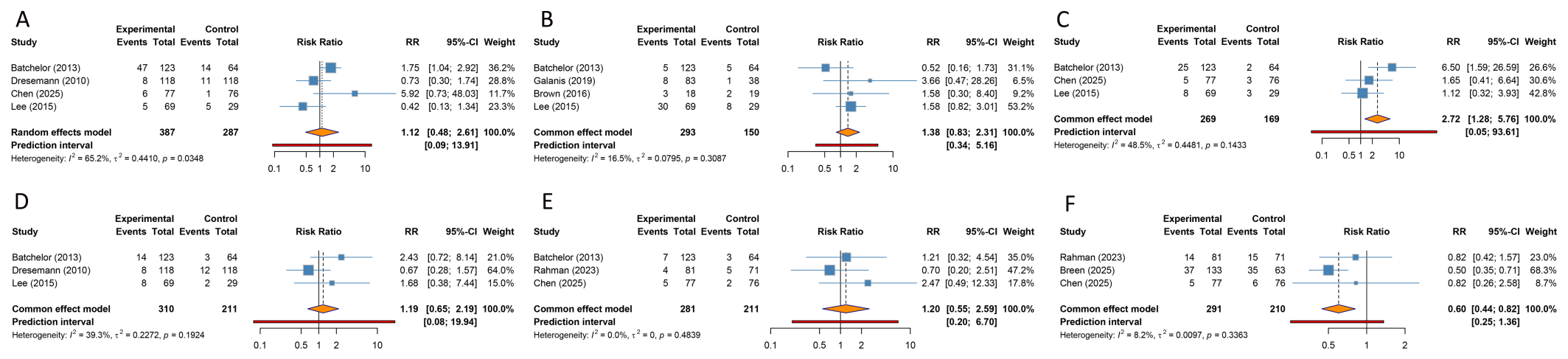


**FIGURE S11** Forest plots of other common grade ≥ 3 adverse events. (A) Alanine aminotransferase increased; (B) Fatigue; (C) Hypertension; (D) Diarrhea; (E) Headache.


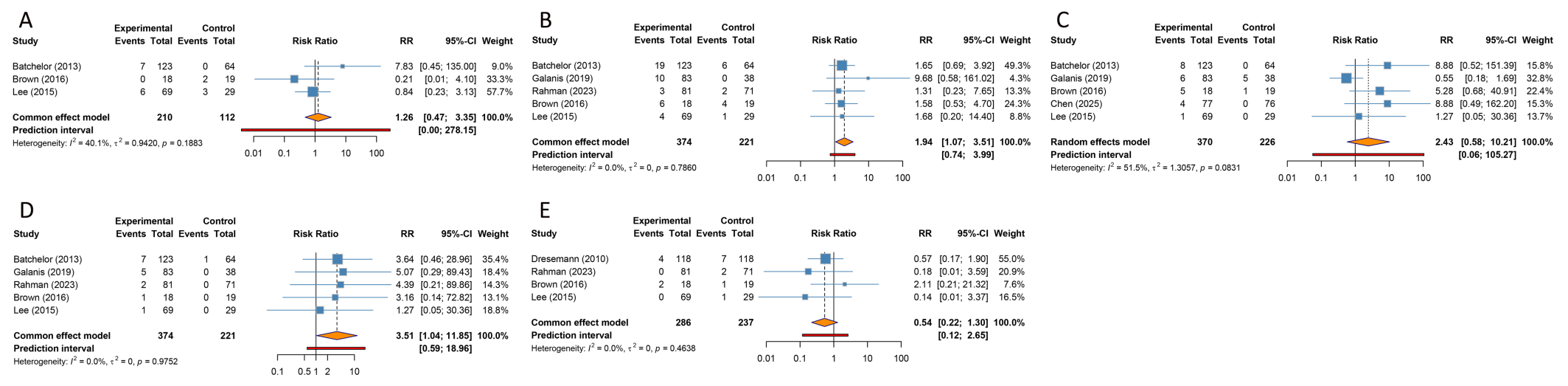


**FIGURE S12** Sensitivity analysis of tyrosine kinase inhibitor combination therapy for glioblastoma. (A) Progression-free survival; (B) Overall survival; (C) Objective response rate; (D) Grade ≥ adverse events.


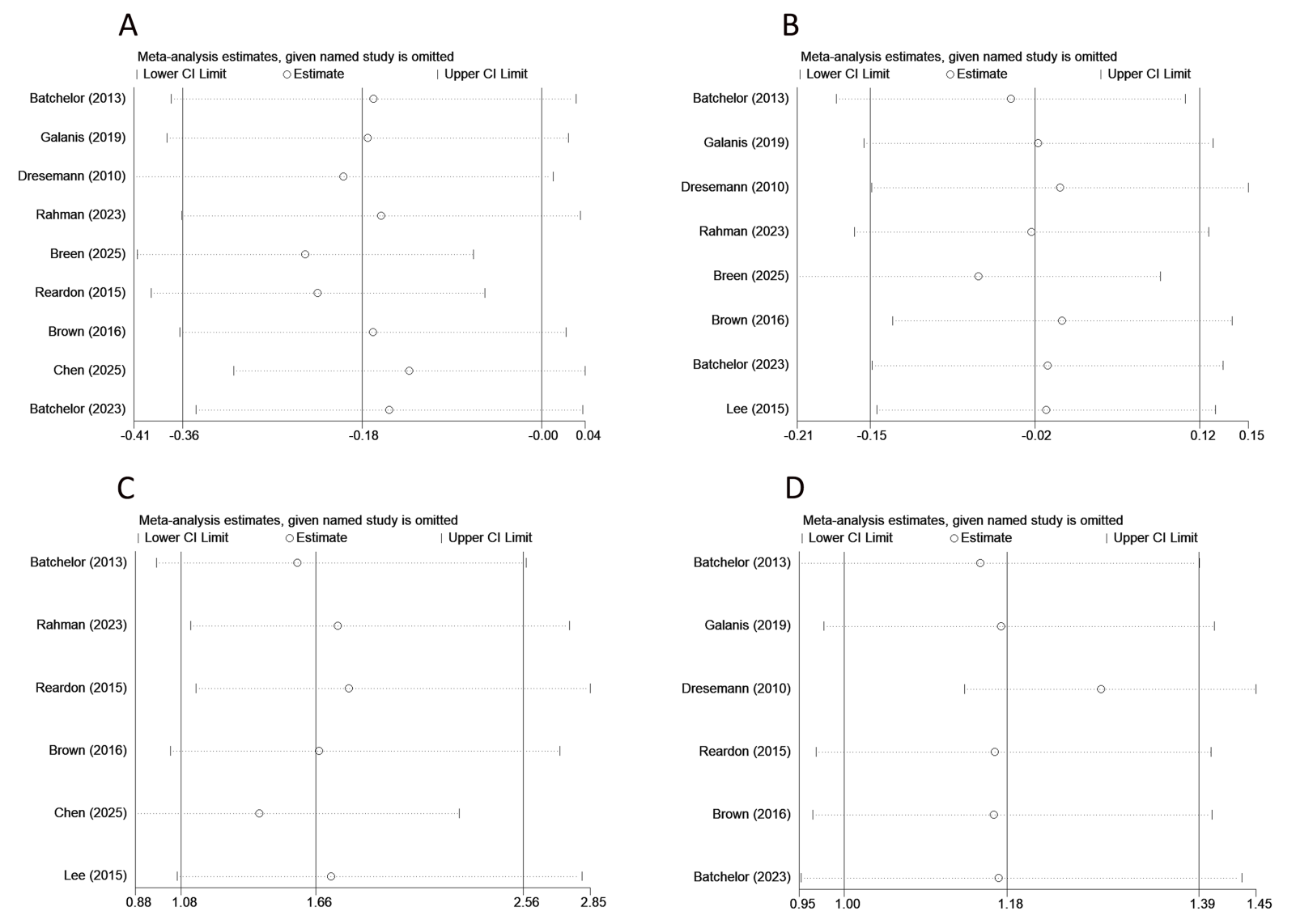


**FIGURE S13** Funnel plots of tyrosine kinase inhibitor combination therapy for glioblastoma. (A) Progression-free survival; (B) Overall survival; (C) Objective response rate; (D) Grade ≥ adverse events.


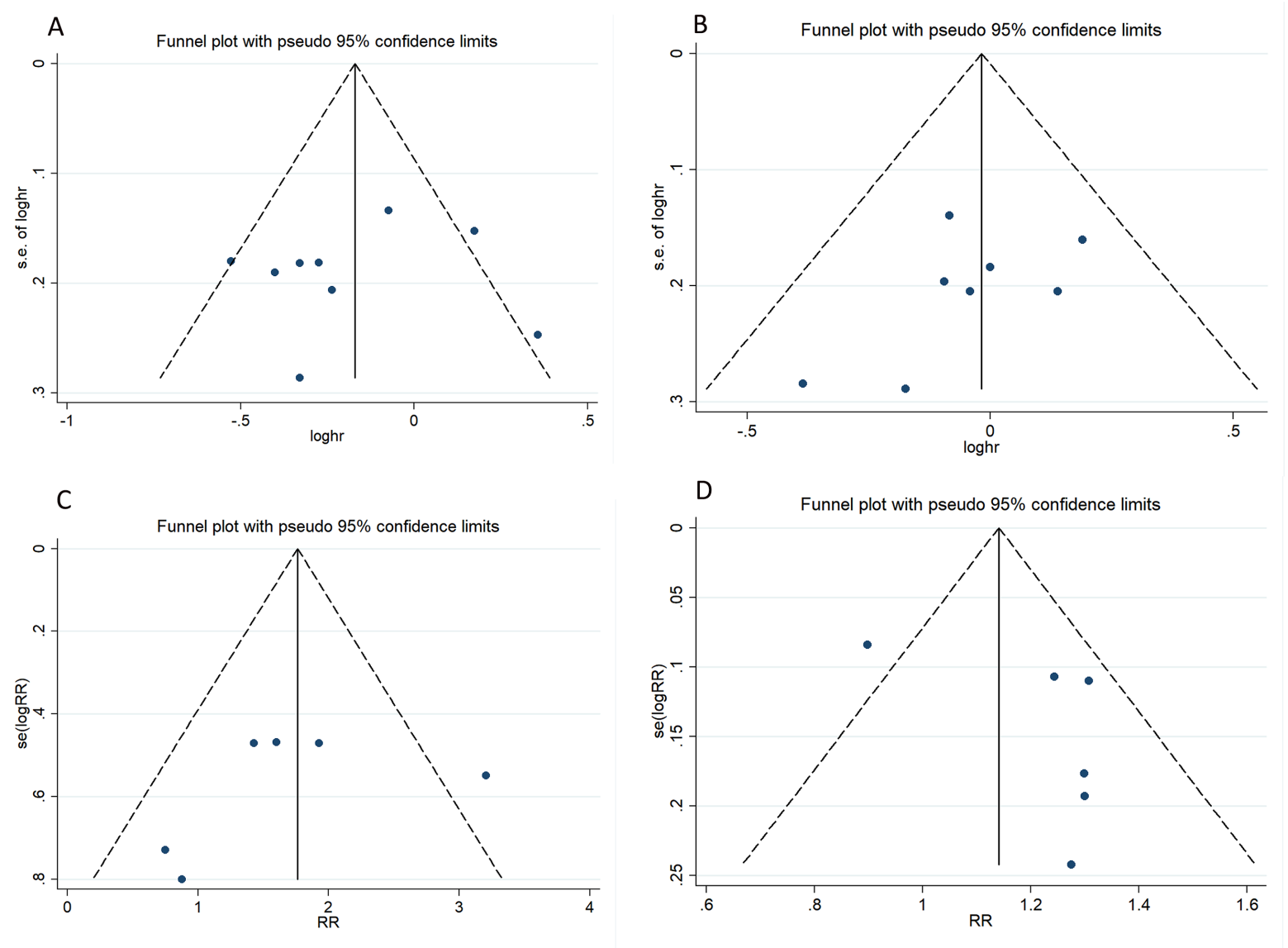

Supplement: Supplementary file 2 [file DataSheet2.docx]
