# Supplementary material for: Efficacy and safety of tyrosine kinase inhibitor combination therapy for glioblastoma: a meta-analysis with trial sequential analysis of randomized controlled trials
Source: Front Oncol. 2026 Apr 20;16:1796708. doi: 10.3389/fonc.2026.1796708 (PMC13135965; doi:10.3389/fonc.2026.1796708)
Supplement: Supplementary file 3 [file Table1.docx]

| **TABLE S1** Quality analysis of the included RCTs by modified Jadad scale. | | | | | | |
| --- | --- | --- | --- | --- | --- | --- |
| Study | Randomization | Randomization concealment | Double blind | Withdrawals and dropouts | Score | Study quality |
| Batchelor et al. (2013) | 2 | 2 | 2 | 1 | 7 | High |
| Galanis et al. (2019) | 1 | 1 | 2 | 1 | 5 | High |
| Rahman et al. (2023) | 2 | 2 | 0 | 1 | 5 | High |
| Dresemann et al. (2010) | 1 | 1 | 0 | 1 | 3 | Low |
| Breen et al. (2025) | 2 | 2 | 2 | 1 | 7 | High |
| Reardon et al. (2015) | 1 | 1 | 0 | 1 | 3 | Low |
| Brown et al. (2016) | 2 | 2 | 2 | 1 | 7 | High |
| Chen et al. (2025) | 2 | 2 | 2 | 1 | 7 | High |
| Batchelor et al. (2023) | 2 | 1 | 2 | 1 | 6 | High |
| Lee et al. (2015) | 1 | 1 | 0 | 1 | 3 | Low |
